# *Plasmodium* protein kinase 2 is required for ookinete-to-oocyst transition and parasite transmission

**DOI:** 10.26508/lsa.202603863

**Published:** 2026-10-05

**Authors:** Sarah L Pashley, Molly Hair, Chiamaka V Ukegbu, Mohammad Zeeshan, Akancha Mishra, Declan Brady, Robert Markus, Sue Vaughan, Carla Pasquarello, Anthony A Holder, Alexandre Hainard, George K Christophides, Dina Vlachou, Pushkar Sharma, David S Guttery, Rita Tewari

**Affiliations:** 1 School of Life Sciences, University of Nottingham , Nottingham, UK; 2 Department of Biological Sciences, University of Oxford Brookes , Oxford, UK; 3 Department of Life Sciences, Imperial College London , London, UK; 4 Proteomics Core Facility, Faculty of Medicine, University of Geneva , Switzerland; 5 Malaria Parasitology Laboratory, The Francis Crick Institute, London, UK; 6 National Institute of Immunology, New Delhi, India

## Abstract

*Plasmodium* parasites rely on diverse protein kinases to regulate critical processes, yet many remain uncharacterised. Protein kinase 2 (PK2) is required for asexual blood-stage proliferation and merozoite invasion, but its role in sexual stages and transmission is unknown. Here, using live-cell and super-resolution imaging, ultrastructural analysis, functional phenotyping, and quantitative phosphoproteomics, we define the role of PK2 in *Plasmodium berghei* transmission. PK2 was expressed in merozoites, ookinetes, and sporozoites: the three invasive forms of the parasite. Conditional depletion of PK2 impaired ookinete motility, disrupted apical microneme positioning, abolished oocyst formation, and prevented mosquito-to-host transmission. Direct haemocoel injection of mutant ookinetes failed to restore sporozoite production, demonstrating an additional requirement for PK2 downstream of midgut traversal. Although global protein abundance was largely preserved, phosphoproteomic profiling identified altered phosphorylation of proteins associated with cytoskeletal organisation, host–cell interaction, midgut invasion, and gene regulation. These findings highlight PK2’s critical role across multiple invasive stages, advancing understanding of malaria parasite transmission and identifying putative PK2-regulated pathways as potential targets for transmission-blocking interventions.

## Abstract (precis)

PK2 is required for *Plasmodium berghei* oocyst development and transmission. PK2 depletion impairs ookinete motility and apical microneme organisation, prevents oocyst formation and subsequent sporozoite production, and alters phosphorylation of proteins associated with cytoskeletal organisation, invasion, and host interaction.

## Introduction

Malaria is a major global public health burden, with an estimated 263 million cases and 597,000 deaths in 2023, mainly children under 5 yr old (World malaria report, 2024). Despite the availability of multiple antimalarial therapies that target the *Plasmodium* parasite, the continued emergence and spread of drug-resistant parasites threatens control and elimination efforts (1). Hence, the identification of novel therapeutic targets and parasite-selective drugs remains a priority. Protein kinases (PKs) regulate signalling networks fundamental to parasite proliferation, development, and transmission, and therefore have attracted considerable interest as such targets (2).

The *Plasmodium* life cycle includes asexual replication in the mammalian host and sexual development within the mosquito vector. Developmental transitions involve extensive genome replication by both mitosis and meiosis, and the generation of specialised invasive cells (3, 4, 5, 6). Three morphologically distinct invasive forms: merozoites, ookinetes, and sporozoites, are responsible for infection, replication, and transmission in mammalian host and mosquito vector stages (3, 7, 8). After sporozoite injection by an infected mosquito, parasites multiply in liver hepatocytes to produce merozoites, which invade erythrocytes. Cyclic propagation of merozoites and invasion of new erythrocytes during asexual blood-stage schizogony (6) result in the pathogenesis associated with the disease malaria. A subpopulation of cells differentiates into gametocytes in the bloodstream, and after their ingestion by a mosquito in a blood meal, these gametocytes are activated and undergo gametogenesis. After gamete fertilisation, the zygote differentiates (over VI stages (3, 9)) into an ookinete in the lumen of the mosquito midgut. The ookinete is a highly motile and invasive cell that traverses the midgut epithelium and establishes an oocyst. Over ∼2–3 wk, the oocyst undergoes prolonged endomitosis (termed sporogony) to produce another invasive stage: the sporozoite (10). Sporozoites migrate to the mosquito salivary glands and are transmitted to the mammalian host in a blood meal. Host-cell or tissue invasion by merozoites, ookinetes, and sporozoites is facilitated by the polarised morphology of the cell, an actomyosin motor, and an apical secretory apparatus variously comprised of micronemes, rhoptries, and dense granules, which are subcellular organelles that secrete proteins required for motility, attachment, and host-cell entry (3).

PKs coordinate signalling pathways in parasite differentiation and host-cell invasion, and yet, a substantial fraction of the *Plasmodium* kinome is poorly characterised. *Plasmodium* spp. encode ∼60–90 PKs (depending on the species (11, 12)), many of which are highly divergent from their human orthologues, supporting their potential as tractable targets for therapeutic intervention (2, 13). A systematic functional analysis of the *Plasmodium berghei* kinome identified 66 PKs, and of these, 23 were dispensable for blood-stage proliferation in the mammalian host. Of these 23, 13 were essential for sexual stages and transmission through the mosquito. The remaining PKs were either redundant or had essential roles at other stages of parasite development in the mammalian host (11).

Here, we focus on protein kinase 2 (PK2), a kinase previously identified as essential for blood-stage development in *P. berghei* and *P. falciparum* (11, 14). Reports differ regarding whether *P. falciparum* (Pf) PK2 is activated by Ca^2+^/calmodulin in vivo (15, 16), but it shares ∼55% sequence similarity with human CaMKIδ. PfPK2 is required for microneme secretion in merozoites (15), and in conditional PfPK2 knockdown parasites, the microneme protein apical membrane antigen 1 (AMA1) fails to translocate to the merozoite surface after parasite egress from the infected erythrocyte, remaining within micronemes. Consequently, merozoites attach to erythrocytes but are unable to complete invasion, and AMA1 abundance in culture supernatants is markedly reduced (15).

Although PK2 function in asexual blood stages has been defined, any role during sexual development and parasite transmission is currently unknown. Using the rodent malaria parasite *P. berghei*, we show that PbPK2 is expressed in two additional invasive forms: ookinetes and sporozoites. Conditional depletion of PbPK2 using a gene promoter-swap strategy resulted in ablation of oocyst formation and blocked parasite transmission. This phenotype was associated with defects in the transition from ookinete to oocyst, decreased apical positioning of micronemes, and impaired ookinete motility required for invasion of the mosquito midgut epithelium.

## Results

### PbPK2 localises to discrete foci throughout asexual blood-stage development

**Figure S1.**
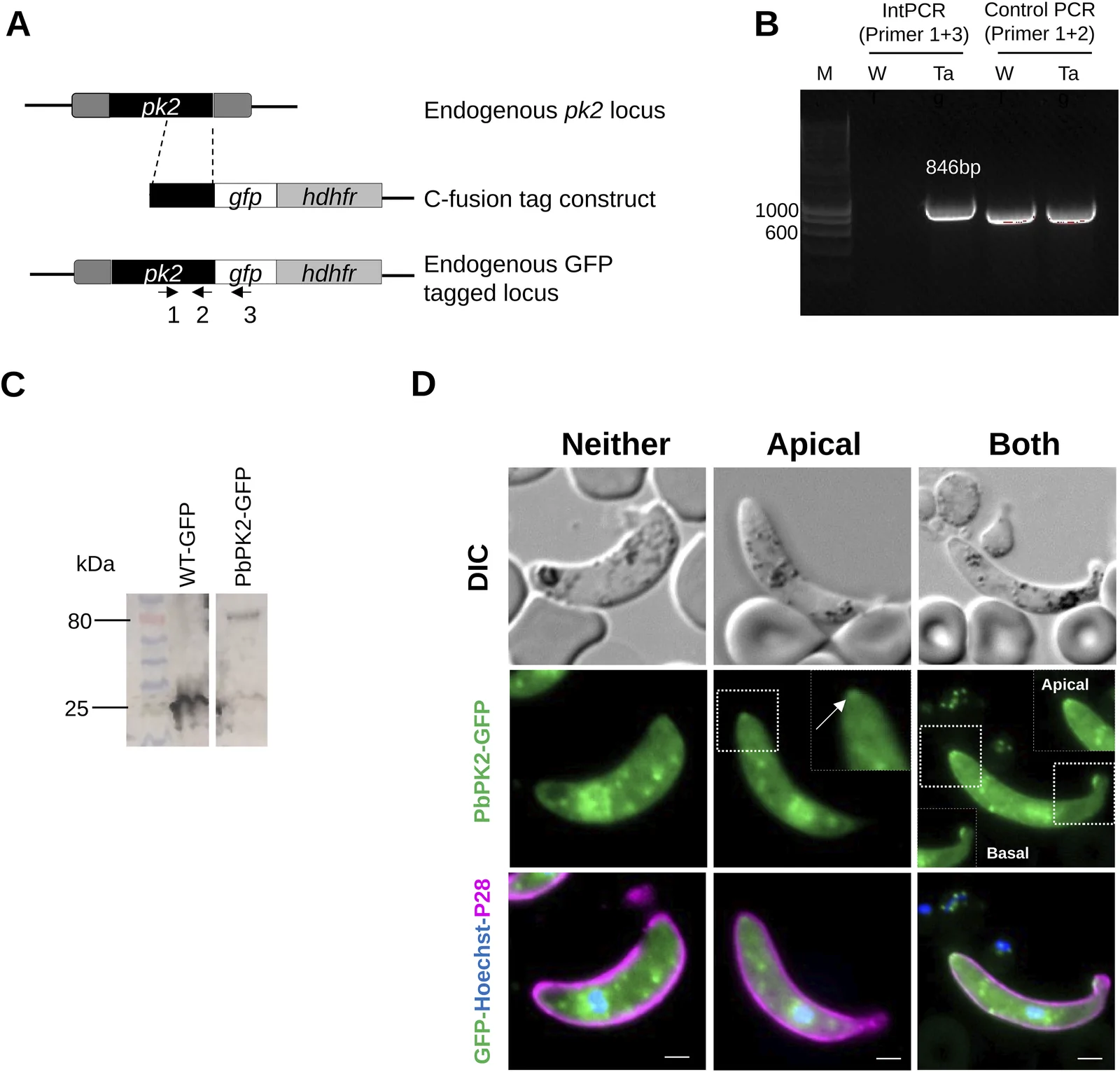
Generation and characterisation of PbPK2-GFP construct and transgenic line. **(A)** Schematic demonstrating single homologous recombination strategy to make PbPK2-GFP using the *pk2* locus and a GFP tagging construct. Arrows represent the position of the primers used to confirm integration. **(B)** Diagnostic PCR to confirm correct integration of *pk2* into the GFP construct. **(C)** Western blot to confirm the molecular weight of the GFP-tagged PK2 protein (molecular weight ∼80 kD) using an antibody against GFP. WT GFP was used as a control (molecular weight ∼25 kD). This Western blot has been spliced to remove other samples that were run on the same gel. Original blot can be found in Source Data. **(D)** Representative examples of PbPK2-GFP fluorescence in stage VI ookinetes. Panels are GFP fluorescence (PbPK2-GFP), 13.1 antibody staining for P28 (P28), and merge of GFP-Hoechst-P28. Scale bars, 2 μm. Neither: no distinct GFP expression at the apical or basal end; apical: GFP fluorescence localised only at apical end; both: GFP fluorescence localised to apical and basal ends. Source data are available for this figure.

**Figure 1.**
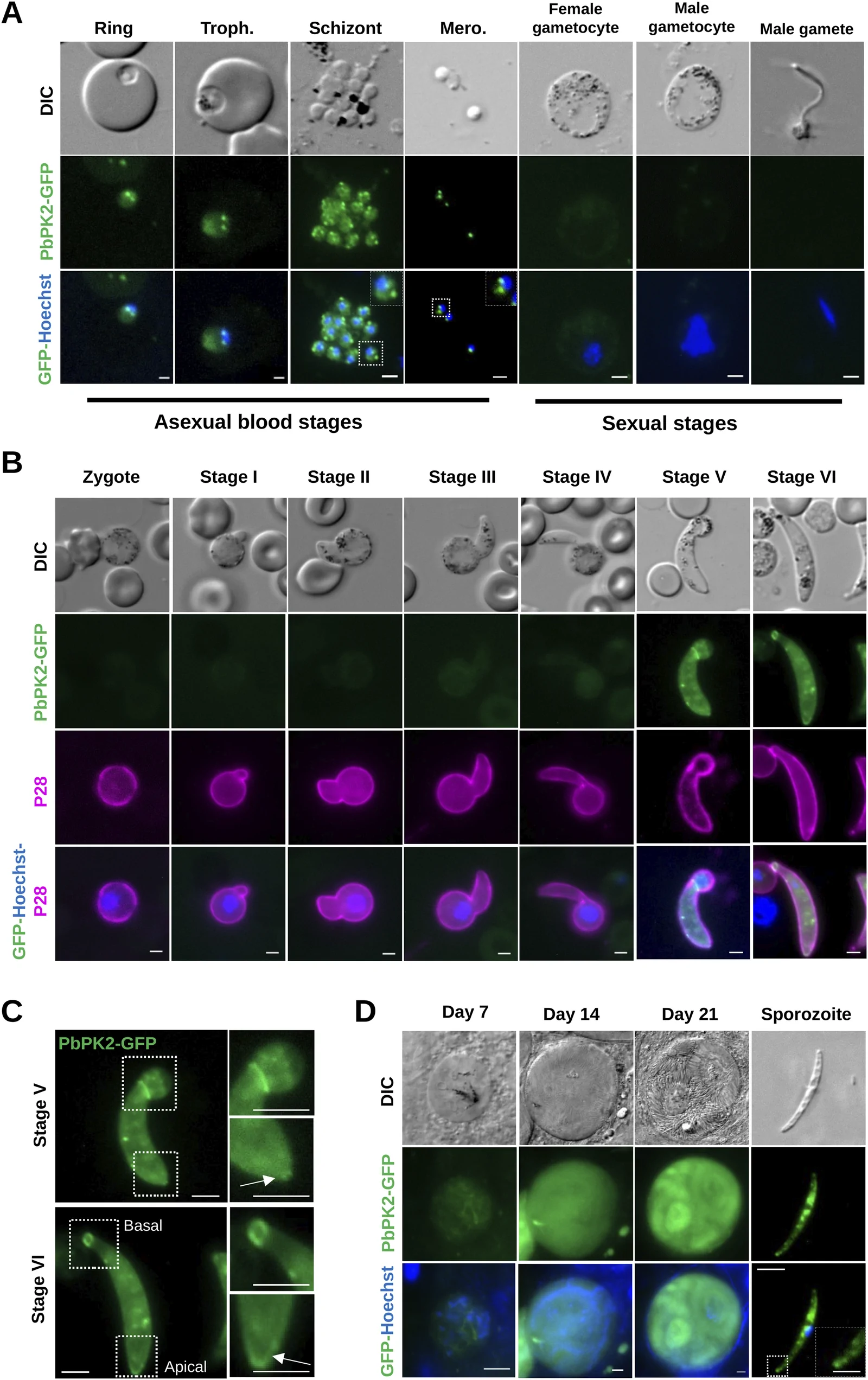
PbPK2-GFP is expressed in all invasive stages of the *P. berghei* life cycle. **(A)** Live-cell imaging of PbPK2-GFP in asexual blood-stage parasites: ring, trophozoite, schizont, and merozoite stages, and sexual-stage gametocytes. Panels are DIC, GFP fluorescence (PbPK2-GFP), and PbPK2-GFP merged with Hoechst staining for nuclei (GFP-Hoechst). Zooms of individual stages are indicated by white boxes. Scale bars, 2 µm. **(B)** Live-cell imaging of PbPK2-GFP during all stages of zygote–ookinete transition. Panels are DIC, GFP fluorescence (PbPK2-GFP), 13.1 antibody staining for P28 (P28—magenta), and merge of GFP-Hoechst-P28. Scale bars, 2 μm. **(C)** PbPK2-GFP fluorescence imaging of stage V and stage VI ookinetes. Scale bars, 2 μm. Arrow indicates enrichment of GFP fluorescence at the apical tip. **(D)** Live-cell imaging of PbPK2 at days 7, 14, and 21 of oocyst development, and sporozoites were isolated from salivary glands 21 d post-infection. Panels are DIC, GFP fluorescence (PbPK2-GFP), and PbPK2-GFP merged with Hoechst staining for nuclei (GFP-Hoechst). Scale bar, 5 μm. Inset scale bar, 2 μm. DIC, differential interference contrast.

To assess the expression and subcellular location of PbPK2, we generated a PbPK2-GFP transgenic *P. berghei* line with GFP fused in-frame to the C terminus of PbPK2 (PBANKA_1453400), expressed from the endogenous *pk2* locus (Fig S1A). Successful genomic integration of GFP sequence was confirmed by diagnostic PCR (Fig S1B), and Western blot confirmed expression of an ∼85kD tagged protein of the predicted size in schizont lysates (Fig S1C). Using this parasite line, first we examined the expression and location of PbPK2-GFP in asexual blood stages using live-cell fluorescence imaging. PbPK2-GFP formed discrete puncta, often including two prominent foci, with at least one clear focus in proximity to the nucleus in all stages (ring, trophozoite, schizont, and merozoite) (Fig 1A).

### PbPK2-GFP is expressed at various stages within the mosquito vector, with a single focus at the apical and basal ends of ookinete stages

We examined PbPK2-GFP in mosquito-stage parasites by live-cell imaging to look for expression in the sexual stages of the life cycle, particularly the invasive ookinetes and sporozoites. No detectable GFP signal was observed in either male or female gametocytes (Fig 1A), or in zygotes and during early ookinete differentiation (stages I–IV) (Fig 1B). At stage V, PbPK2-GFP became readily detectable throughout the developing ookinete, with a broadly distributed intracellular signal together with several regions of focal enrichment. These included prominent internal foci, frequently in proximity to the nucleus, as well as enrichment at the apical and basal ends in a subset of parasites (Figs 1B and C and S1D). A structure resembling the developing contractile ring was also apparent in some cells. In mature ookinetes (stage VI), PbPK2-GFP remained distributed throughout the parasite body with multiple discrete intracellular foci. Of 53 ookinetes imaged, 36% (19/53) exhibited PbPK2-GFP fluorescence only at the apical end, 45% (24/53) exhibited PbPK2-GFP fluorescence at both the apical and basal ends, and 19% (10/53) did not exhibit enrichment at either end (representative examples in Fig S1D).

To investigate PbPK2-GFP expression during sporogony, we allowed *Anopheles stephensi* mosquitoes to feed on mice infected with PbPK2-GFP parasites and analysed oocyst development on day 7, 14, and 21 post-blood meal. PbPK2-GFP was barely detectable in day 7 oocysts (Fig 1D) but was detectable in day 14 oocysts during sporulation (Fig 1D). In contrast, PK2-GFP was strongly expressed in fully sporulated oocysts at day 21 (Fig 1D). We also isolated sporozoites from salivary glands for imaging at day 21 post-feeding. PK2-GFP was detectable in salivary gland sporozoites (Fig 1D), with a dispersed intracellular location and multiple foci but with consistent enrichment at the apical tip in some cells, mirroring the pattern observed in ookinetes. Collectively, these GFP fluorescence data indicate that PK2-GFP is expressed in the invasive mosquito stages and displays a broadly distributed, punctate localisation pattern with variable polar enrichment.

### Super-resolution imaging (structured illumination microscopy) reveals subcellular distribution of PK2 in schizonts and enrichment at the apical and basal ends of ookinetes

**Figure 2.**
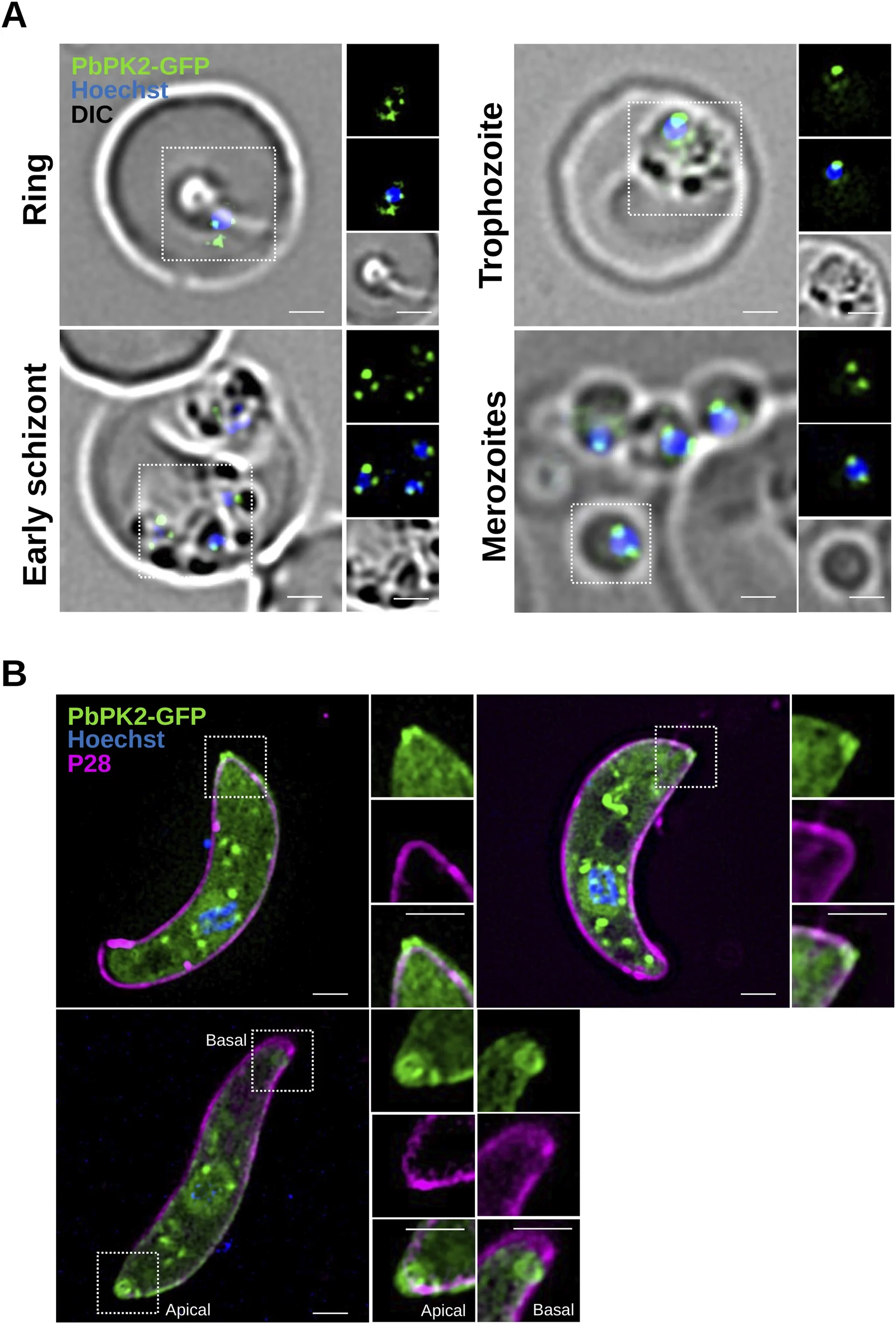
Location of PbPK2-GFP in invasive parasite stages revealed using SIM. **(A)** SIM imaging of PbPK2-GFP asexual-stage parasite (ring, trophozoite, schizont, and merozoites). Panels are DIC, GFP fluorescence (PbPK2-GFP), and PbPK2-GFP merged with Hoechst staining for nuclei (GFP-Hoechst). Scale bars, 2 μm. **(B)** SIM imaging of PbPK2-GFP ookinetes. Panels are GFP fluorescence (PbPK2-GFP), 13.1 antibody staining for P28 (P28—magenta), and merge of GFP-Hoechst-P28. Scale bars, 2 μm. DIC, differential interference contrast.

To define the subcellular distribution of PbPK2 at higher spatial resolution, we examined PbPK2-GFP parasites using structured illumination microscopy (SIM). In blood-stage parasites, PbPK2-GFP was observed as discrete foci adjacent to the nucleus throughout ring, trophozoite, early schizont, and merozoite stages (Fig 2A). In early schizonts, multiple PbPK2-GFP puncta were observed outside the Hoechst-stained nuclei, consistent with an extranuclear localisation. PbPK2-GFP foci were also retained in individual merozoites, indicating that the punctate distribution of PbPK2 persists during schizogony and merozoite formation.

We next used SIM to examine PbPK2-GFP localisation in mature ookinetes stained for the surface protein P28. PbPK2-GFP displayed a punctate distribution throughout the ookinete, including multiple internal foci frequently concentrated around the nucleus, together with regions of enrichment near the cell periphery and at the poles (Fig 2B). PbPK2-GFP was enriched at the apical end and formed a ring structure in proximity with the conoid body, and in a subset of cells, it also formed a ring-like structure at the basal end, indicative of an association with the basal complex (Fig 2B). As with live-cell imaging, these patterns were not observed in all ookinetes, suggesting that polar enrichment of PbPK2 may be transient or restricted to particular cellular states. Together, these observations show that PbPK2 is organised into discrete foci in schizonts and ookinetes, with enrichment at both the apical and basal ends of mature ookinetes.

**Figure S2.**
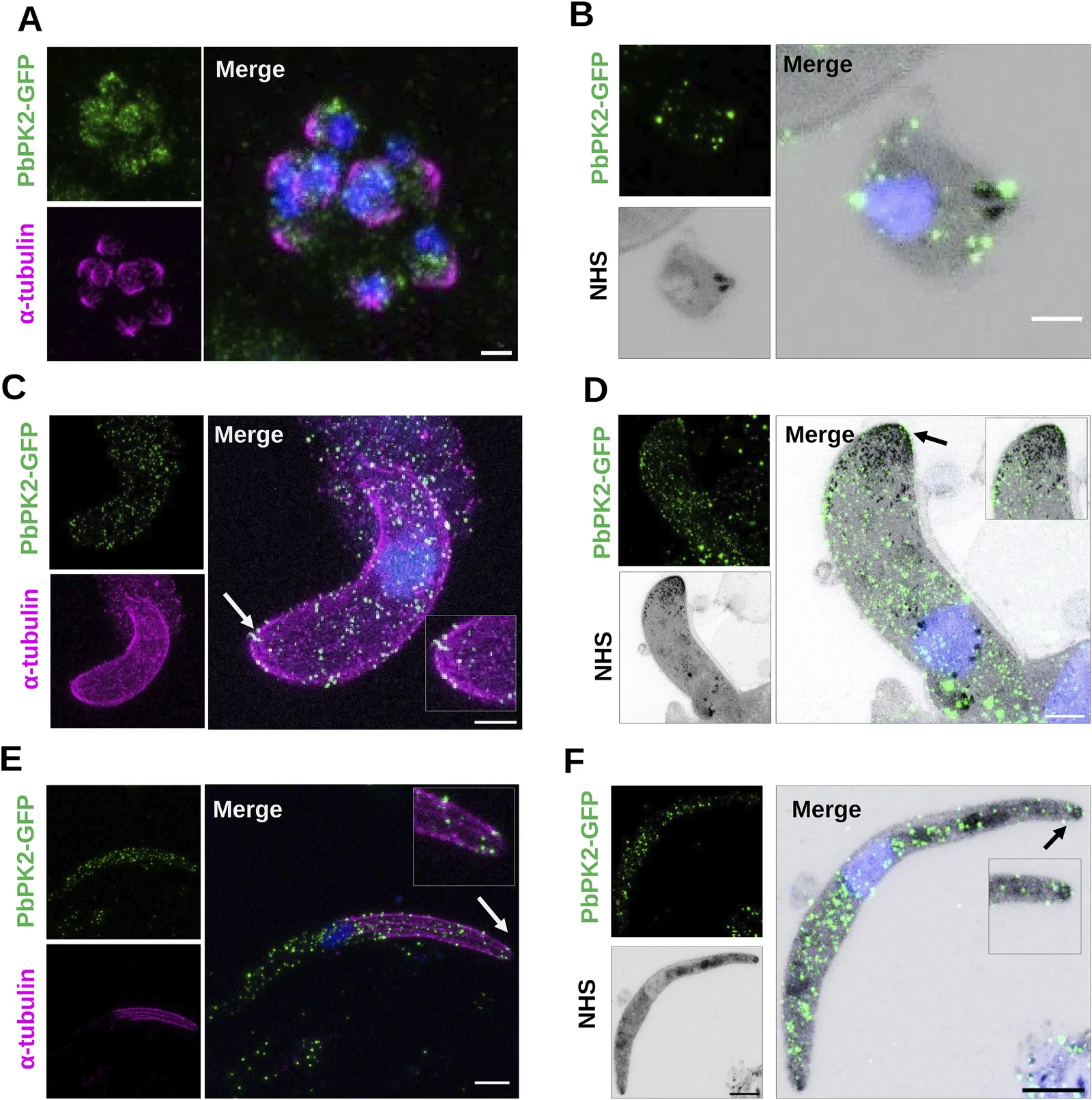
U-ExM of PbPK2-GFP invasive-stage parasites. **(A)** U-ExM imaging of a PbPK2-GFP schizont labelled with GFP (green) and with α-tubulin (magenta). The merge panel also includes Hoechst staining for nuclei. Scale bar, 2 μm. **(B)** U-ExM imaging of PbPK2-GFP merozoites labelled with GFP (green) and with NHS-ester (grey). In the merge panel, the two signals are merged with Hoechst DNA staining (blue). Scale bar, 2 μm. **(C)** U-ExM imaging of a PbPK2-GFP ookinete labelled with GFP (green) and with α-tubulin (magenta). The merge panel also includes Hoechst staining for nuclei. The white arrow indicates the apical end of the cell, and the white box indicates the zoomed area within the image. Scale bar, 2 μm. **(D)** U-ExM imaging of PbPK2-GFP ookinetes labelled with GFP (green) and with NHS-ester (grey). In the merge panel, the two signals are merged with Hoechst DNA staining (blue). The black arrow indicates the apical end of the cell, and the black box indicates the zoomed area within the image. Scale bar, 2 μm. **(E)** U-ExM imaging of a PbPK2-GFP salivary gland sporozoite labelled with GFP (green) and with α-tubulin (magenta). In the merge panel, the two signals are merged with Hoechst DNA staining (blue). The white arrow indicates the apical end of the cell, and the white box indicates the zoomed region within the image panel. Scale bar, 2 μm. **(F)** U-ExM imaging of a PbPK2-GFP salivary gland sporozoite labelled with GFP (green) and with NHS-ester (grey). In the merge panel, the two signals are merged, together with Hoechst DNA staining (blue). The black arrow indicates the apical end of the cell, and the black box indicates the zoomed area within the image. Scale bar, 2 µm.

We next attempted to complement our live-cell and SIM findings using ultrastructure expansion microscopy (U-ExM) of invasive stages (merozoites, ookinetes, and sporozoites), staining for tubulin and GFP to assess the proximity of PbPK2-GFP to microtubules, and NHS-ester for labelling of prominent subcellular apical structures such as rhoptries and micronemes that are involved in invasion. However, although GFP signal appeared dispersed throughout the parasite at all stages, definitive PbPK2-GFP location to microtubules or subcellular structures at any stage was inconclusive (Fig S2A–F).

### Conditional knockdown of PbPK2 revealed a role for PK2 in the ookinete-to-oocyst transition and parasite transmission

**Figure S3.**
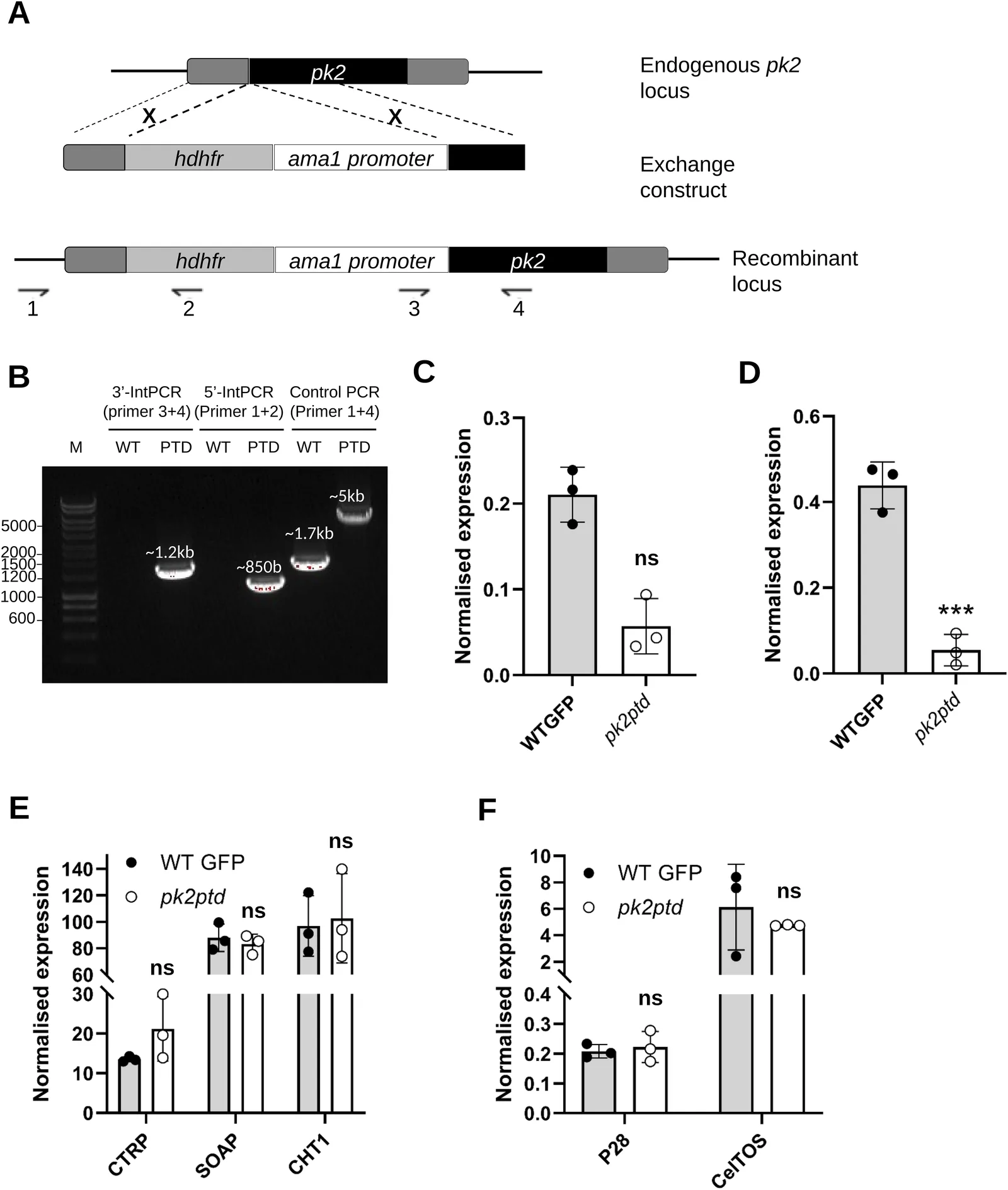
Generation and characterisation of conditional knockdown *pk2ptd*. **(A)** Schematic demonstrating double homologous recombination strategy used to swap the promoter of *pk2* for the promoter of *ama1*. Arrows 1 and 2 indicate the position of primers used to confirm 5′ integration and 3 and 4 the position of primers used to confirm 3′ integration. Primer 1 is the integration primer that anneals from the genomic sequence outside of the homology arm of the plasmid, thereby indicating correct integration. **(B)** Diagnostic PCR was performed to confirm correct integration using primers IntPTD 61_5′ and 5′-IntPTD to check the integration of the selectable marker and IntPTD61_3′ and 3′-IntAMA1 to check the integration of the *ama1* promoter. **(C)** qRT–PCR analysis of *pk2* transcript levels in WT GFP and *pk2ptd* gametocytes activated for 15 mins. Dots show means of three independent experiments, each measured in technical duplicate. Horizontal bar = mean of the three biological replicates ±SD. Expression was normalised to the control genes *arginyl-tRNA synthetase* (PBANKA_1434200) and *hsp70* (PBANKA_0818900). ns = not significant. **(D)** qRT–PCR analysis of *pk2* transcript levels in WT GFP and *pk2ptd* ookinetes 24 hpa. Dots show means of three independent experiments, each measured in technical duplicate. Horizontal bar = mean of the three biological replicates ±SD. ***P < 0.001. Expression was normalised to the control genes *arginyl-tRNA synthetase* (PBANKA_1434200) and *hsp70* (PBANKA_0818900). **(E)** qRT–PCR analyses of the transcript levels of highly expressed invasion genes—CTRP, SOAP, and CHT1—in WT GFP and *pk2ptd* ookinetes 24 hpa. Dots show means of three independent experiments, each measured in technical duplicate. Horizontal bar = mean of the three biological replicates ±SD. Expression was normalised to the control genes *arginyl-tRNA synthetase* (PBANKA_1434200) and *hsp70* (PBANKA_0818900). ns = not significant. **(F)** qRT–PCR analyses of the transcript levels of less-expressed invasion genes—CelTOS and P28—in WT GFP and *pk2ptd* ookinetes 24 hpa. Dots show means of three independent experiments, each measured in technical duplicate. Horizontal bar = mean of the three biological replicates ±SD. Expression was normalised to the control genes *arginyl-tRNA synthetase* (PBANKA_1434200) and *hsp70* (PBANKA_0818900). ns = not significant. SOAP, secreted ookinete adhesive protein; CelTOS, cell-traversal protein for ookinetes and sporozoites; CHT1, chitinase; CTRP, circumsporozoite- and thrombospondin-related adhesive protein–related protein.

Our observation that PbPK2-GFP is expressed in invasive ookinete and sporozoite stages in the mosquito led us to examine the function of PK2 in these stages. Using a promoter trap double-crossover strategy, we created a conditional knockdown *P. berghei* mutant (henceforth termed *pk2ptd*) that placed the *pk2* gene under the control of the *ama1* promoter (PBANKA_0915000), which has low transcript levels in *P. berghei* gametocytes (17) (Fig S3A). Correct integration of the *ama1* promoter was confirmed by diagnostic PCR (Fig S3B). Quantitative reverse transcription–PCR (qRT–PCR) confirmed that *pk2* transcript levels were reduced by 66% in activated *pk2ptd* gametocytes and significantly by 87% in *pk2ptd* ookinetes (Fig S3C and D).

**Figure 3.**
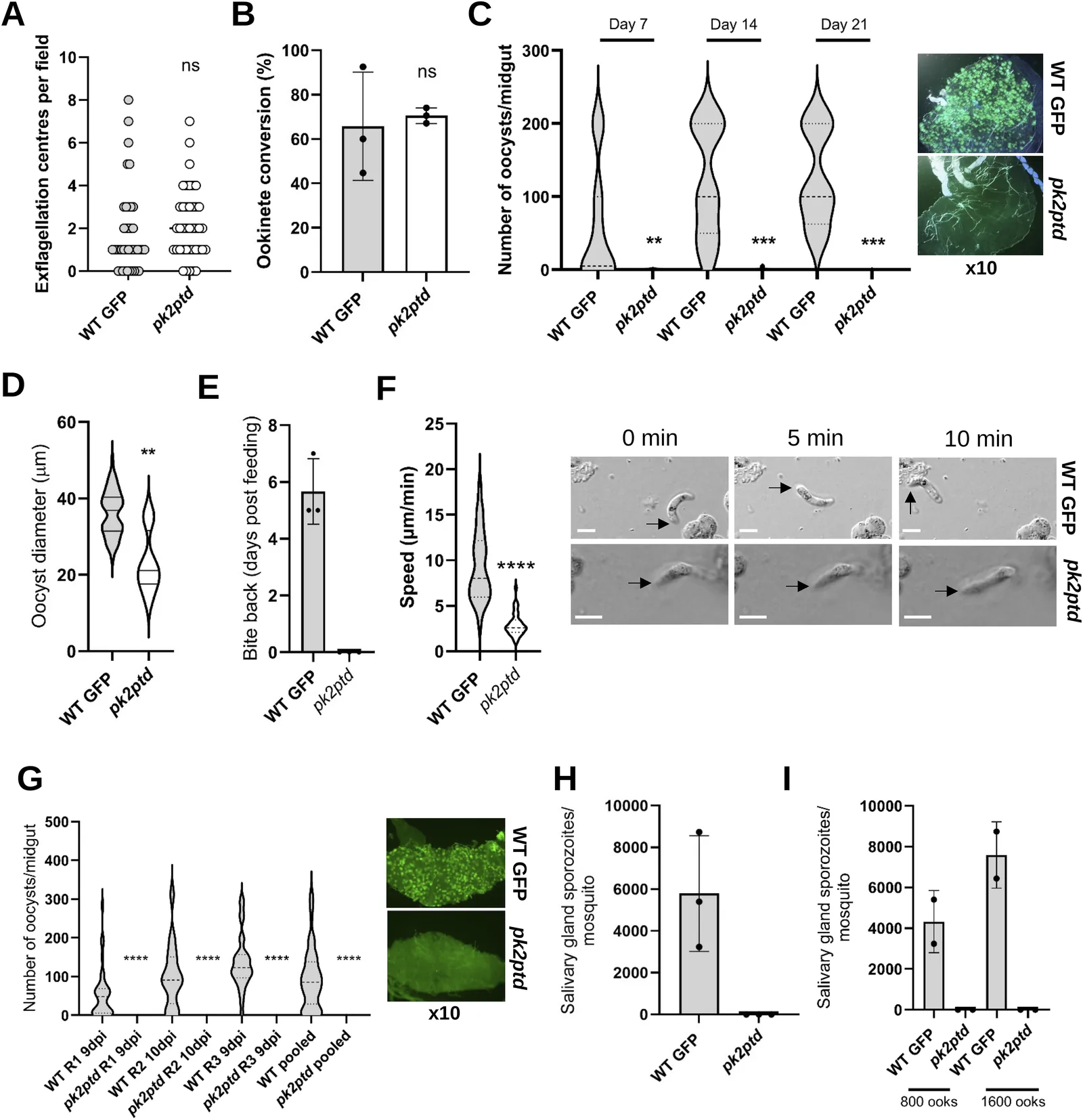
PbPK2 is required for ookinete-to-oocyst transition and parasite transmission. **(A)** Exflagellation centres per field at 15 min post-activation. n = 3 independent experiments (>20 fields per experiment). **(B)** Percentage of ookinete conversion. n = 3 independent experiments (>100 cells). Error bar ± SEM. **(C)** Total number of GFP-positive oocysts per infected mosquito in *pk2ptd* compared with WT GFP parasites at 7, 14, and 21 d post-infection. Violin plots show the data distribution, with the median and interquartile range (25th–75th percentiles) indicated. n = 3 independent experiments. **P < 0.01; ***P < 0.001. Representative images of WT GFP and *pk2ptd* infected midguts at 10× magnification are given to the right of the violin plot. **(D)** Diameter of individual *pk2ptd* and WT GFP oocysts (μm) at 14-d post-infection. Violin plots show the data distribution, with the median and interquartile range (25th–75th percentiles) indicated (60 oocysts quantified across three mosquito infections and 10 guts per experiment for WT GFP, and 11 oocysts quantified over the three experiments for *pk2ptd*). **P < 0.01. **(E)** Bite‐back experiments for transmission of WT GFP and *pk2ptd* parasites from mosquito to mouse. Infected mosquitoes were fed on mice, and then, the mice were monitored for day of patent blood-stage parasitaemia indicative of successful infection. n = three independent experiments, 20 mosquitoes per experiment. **(F)** Velocity of individual WT GFP or *pk2ptd* ookinetes from 24-h cultures measured over 10 min and shown in the violin plot. Violin plots show the data distribution, with the median and interquartile range (25th–75th percentiles) indicated. n = 26 ookinetes for WT GFP and 39 ookinetes for *pk2ptd*, respectively, across two experiments. ****P < 0.0001. Representative frames from time-lapse movies of WT GFP (upper panels) and *pk2ptd* (lower panels) ookinetes in Matrigel at 0, 5, and 10 min. Scale bars = 5 μm. The arrow highlights the apical end of the ookinete. **(G)** Number of GFP-positive oocysts per mosquito infected with *pk2ptd* and WT GFP parasites at 9 or 10 d post-infection. Violin plots show the data distribution, with the median and interquartile range (25th–75th percentiles) indicated. n = 3 independent experiments. ****P < 0.0001. Representative images of midguts infected with WT GFP and *pk2ptd* parasites at 10× magnification are shown. **(H)** Number of sporozoites per mosquito in 21-d post-infection salivary glands for WT GFP and *pk2ptd* parasite lines. Three independent experiments, n = 20 mosquitoes for each replicate. **(I)** Salivary gland sporozoites per mosquito after direct injection of WT GFP or *pk2ptd* ookinetes into the mosquito haemocoel (inocula of 800 or 1,600 ookinetes). Mean ± STD. n = 2 independent experiments per inoculum.

Male gamete production (assessed by the formation of exflagellation centres) was unaffected in the *pk2ptd* line compared with wild-type (WT)-GFP controls (Fig 3A), and fertilisation and zygote-to-ookinete conversion were also unaffected (Fig 3B). However, *pk2ptd* oocyst development in mosquitoes was almost completely ablated. No *pk2ptd* oocysts were observed at day 7 post-infection, only 11 were detected at day 14 (Fig 3C), and only 1 was detected at day 21, respectively, whereas hundreds of oocysts were present at each time point in comparable mosquitoes fed WT GFP parasites. The few *pk2ptd* oocysts were significantly smaller than WT GFP oocysts (Fig 3D). To confirm the absence of any viable oocyst or sporozoite, infected mosquitoes were allowed to feed on naïve mice. Although mosquitoes were able to transmit the WT GFP parasite, and blood-stage infection was observed on average 5 d later (Fig 3E), mosquitoes infected with *pk2ptd* parasites failed to infect susceptible mice.

Because of the absence of oocyst development, we analysed ookinete motility (18) and found that gliding motility in *pk2ptd* ookinetes was significantly reduced compared with that of WT parasites (Fig 3F; Video 1 and Video 2), indicating that their motor function is impaired and suggesting that this defect may be the cause of the ablation of oocyst development in *pk2ptd* lines, potentially by preventing invasion of the midgut epithelium. In an independent insectary (Imperial College), mosquitoes infected with the same lines confirmed ablation of *pk2ptd* oocyst development, with no oocysts detected 9 or 10 d after infection (Fig 3G), and no sporozoites recovered from mosquito salivary glands 21 d post-infection (Fig 3H). In contrast, with the WT GFP control line hundreds of oocysts were detected in mosquitoes (Fig 3G), resulting in several thousand sporozoites per mosquito (Fig 3H).

Video 1 Representative video of WT GFP ookinete motility. Download video

Video 2 Representative video of *pk2ptd* ookinete motility. Download video

To determine whether the lack of *pk2ptd* oocysts and sporozoites was due to a defect in invasion of the midgut epithelium, we bypassed the midgut barrier by injecting either 800 or 1,600 ookinetes directly into the haemocoel of *A. stephensi* mosquitoes and analysed salivary glands for sporozoites 21 d post-injection (18). No sporozoites were observed in mosquitoes infected with *pk2ptd* ookinetes, whereas the WT GFP control line produced several thousand sporozoites per mosquito (Fig 3I), suggesting that although *pk2ptd* ookinete motility is significantly affected and PK2 may have a significant role during ookinete-to-oocyst transition, it potentially has an additional role in sporozoite development independent of ookinete motility.

### Ultrastructural studies of *pk2ptd* ookinetes showed that PK2 is important for microneme formation and distribution

**Figure 4.**
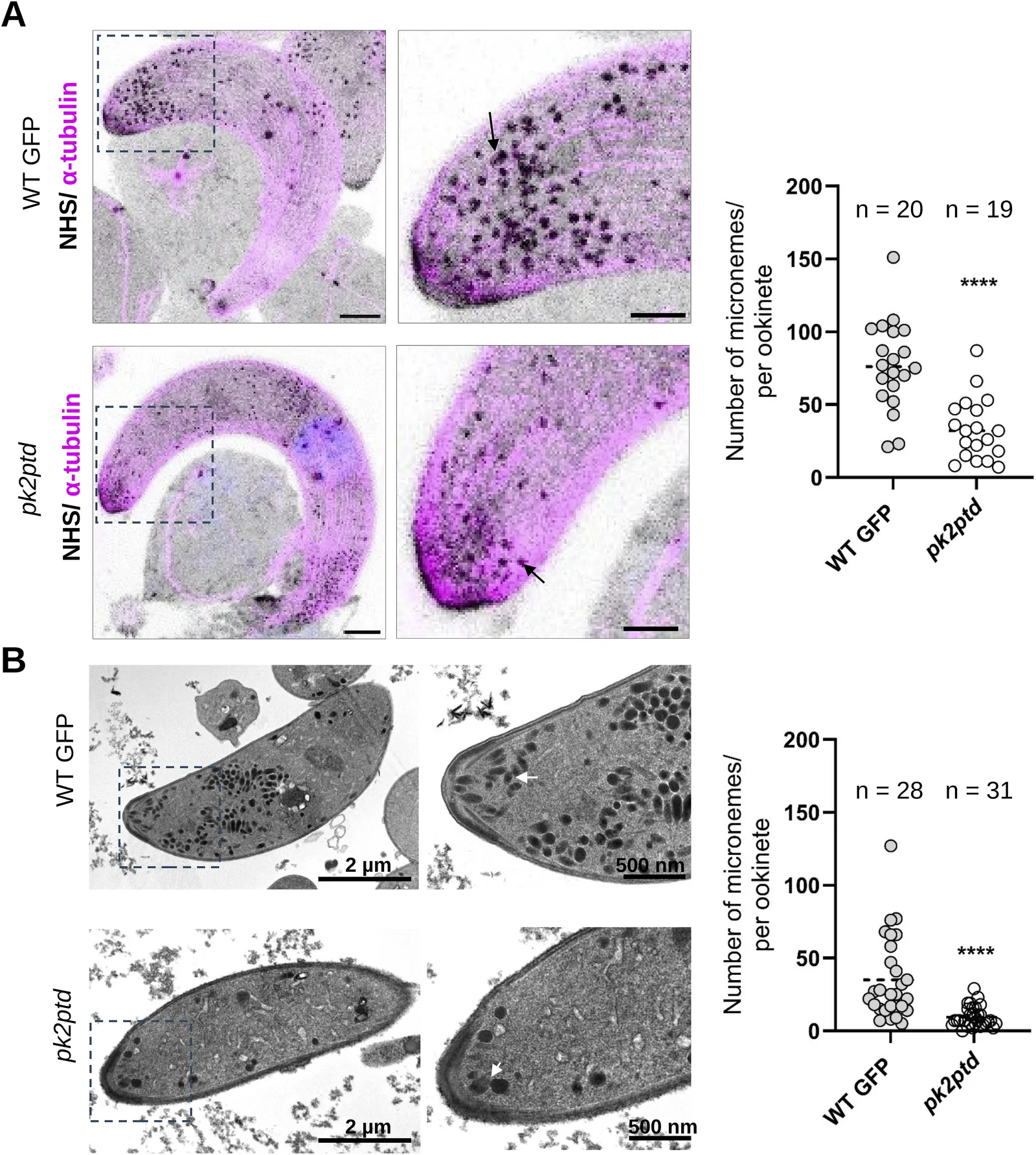
*pk2ptd* ookinetes have altered distribution of apical micronemes. **(A)** Representative U-ExM images of WT GFP and *pk2ptd* ookinetes stained with α-tubulin (magenta), NHS-ester (grey), and Hoechst (blue). Scale bars, 1 μm. Black dashed boxes on the left-hand panels indicated the region of the images shown at higher magnification in the right-hand panels. Scale bars, 500 nm. Right panel: number of micronemes at the apical end in WT GFP (n = 20 cells across two experiments) versus *pk2ptd* ookinetes (n = 19 cells across two experiments) identified by NHS-ester staining and quantified using ImageJ. Micronemes quantified using ImageJ across maximum-intensity projections. ****P < 0.0001. **(B)** TEM imaging of WT GFP and *pk2ptd* ookinetes. The black dashed boxes on the left-hand panels indicate the region of the images shown at higher magnification in the right-hand panels. The scale bar size is indicated in images. Right panel: number of micronemes at the apical end in WT GFP (n = 28 cells across two experiments) versus *pk2ptd* ookinetes (n = 31 cells across two experiments) from TEM images, quantified using ImageJ. ****P < 0.0001.

We used U-ExM and transmission electron microscopy (TEM) to look for ultrastructural defects in *pk2ptd* ookinetes that may be responsible for the impaired invasion phenotype. U-ExM with anti-tubulin antibody and NHS-ester labelling of protein-dense compartments showed *pk2ptd* mature ookinete morphology to be similar to that of the WT GFP control (Fig 4A). In contrast, a quantitative analysis of apical micronemes/granules revealed a significant reduction in *pk2ptd* parasites (mean 32 micronemes across two experimental replicates, n = 19 cells) relative to WT GFP (mean 76 micronemes across two experimental replicates, n = 20 cells, *P* < 0.0001), with microneme-like structures frequently present away from the apex and towards the basal region in *pk2ptd* parasites (Fig 4A). This spatial shift is consistent with a defect in the apical targeting or retention of micronemes during ookinete maturation.

To support these U-ExM observations, we used TEM with both WT GFP and *pk2ptd* mature ookinetes. TEM confirmed that the overall cellular organisation of *pk2ptd* ookinetes was similar to that of WT GFP parasites (Fig 4B) and that there was a significant depletion of apical micronemes (*pk2ptd* mean 9.5 micronemes across two experimental replicates, n = 31 cells, versus WT GFP mean 35.1 micronemes across two experimental replicates, n = 28 cells, *P <* 0.0001), with an increased number of micronemes towards the basal end of *pk2ptd* parasites (Fig 4B).

Because microneme abundance and positioning were altered, we asked whether the transcript levels of key microneme-associated invasion proteins were significantly altered in *pk2ptd* ookinetes. qRT–PCR analysis of 24-h ookinete cultures revealed a modest, but non-significant, increase in circumsporozoite- and thrombospondin-related adhesive protein–related protein (CTRP) transcription, whereas secreted ookinete adhesive protein, chitinase 1 (CHT1), P28, and cell-traversal protein for ookinetes and sporozoites transcript levels were similar in WT GFP and *pk2ptd* parasites (Fig S3E and F). These data suggest that the microneme mislocalisation phenotype of *pk2ptd* ookinetes is unlikely to be driven by a widespread transcriptional down-regulation of genes for canonical microneme cargo.

### Differential phosphorylation of multiple proteins involved in midgut invasion and gene expression in *pk2ptd* parasites

**Figure 5.**
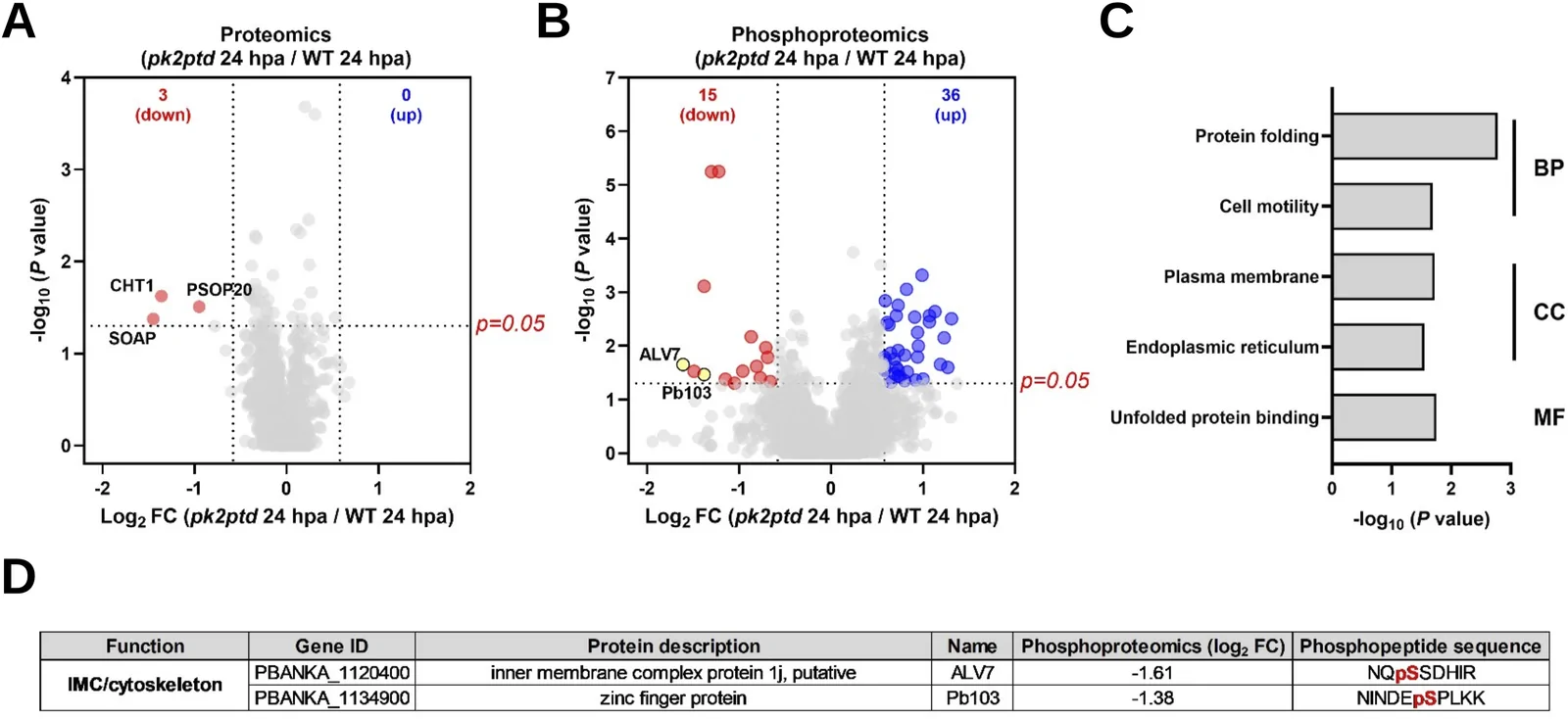
PK2 depletion in ookinetes at 24 hpa has a little effect on total protein abundance but drives changes in phosphorylation. **(A)** Volcano plot comparison of total proteome from *pk2ptd* and WT GFP parasites at 24 hpa. Points are quantified proteins plotted as log_2_ fold change (*pk2ptd*/WT) versus –log_10_ (*P*-value). n = 3 biological replicates for WT GFP and *pk2ptd* lines, respectively; significantly decreased proteins are highlighted (orange) and labelled (SOAP, CHT1, PSOP20). **(B)** Volcano plot comparison of phosphoproteome changes at 24-hpa ookinetes from *pk2ptd* and WT GFP lines. n = 3 biological replicates for WT GFP and *pk2ptd* lines, respectively. Phosphosites significantly decreased (red) or increased (blue) in abundance are indicated, with examples labelled in yellow. **(C)** Gene Ontology analysis of altered phosphoproteins performed using PlasmoDB Gene Ontology slim terms (BP: Biological Process, CC: Cellular Component, MF: Molecular Function). **(D)** Summary table of selected differentially phosphorylated proteins grouped by function, showing gene ID, protein annotation, log_2_ fold change, and corresponding phosphopeptide sequence. Vertical and horizontal dashed lines in (A, B) indicate fold change and significance thresholds used for hit calling. CHT1, chitinase; SOAP, secreted ookinete adhesive protein; PSOP20, putative secreted ookinete protein 20.

To examine how a reduction in *pk2* transcripts impacts total protein abundance and phosphorylation in mature ookinetes, we performed quantitative phosphoproteomic analysis (with phosphopeptide enrichment) comparing WT GFP and *pk2ptd* parasites at 24 h post-activation (hpa) (Fig 5). Using thresholds of log_2_ FC ≥0.58 or ≤−0.58 and *P* ≤ 0.05, the proteomics analysis revealed few changes in overall protein abundance, with only three proteins significantly reduced and none significantly increased in *pk2ptd* parasites (Fig 5A and Table S1). Secreted ookinete adhesive protein, CHT1, and putative secreted ookinete protein 20 were decreased in the *pk2ptd* line. These data indicate that the ookinete proteome at 24 hpa is largely preserved, despite the depletion of PK2. In contrast, the phosphoproteomic analysis identified significant changes in 51 phosphopeptides in *pk2ptd* parasites (15 decreased; 36 increased; Fig 5B and Table S2). These sites of differential phosphorylation mapped to proteins important in protein folding and cell motility (Fig 5C). Notably, several IMC/cytoskeletal-associated/crystalloid-associated proteins had reduced phosphorylation in *pk2ptd* parasites, including the alveolin, ALV7, and the zinc finger protein Pb103 (Fig 5D). Together, these data indicate that *pk2ptd* parasites with depleted PK2 have significantly altered phosphorylation of proteins important in ookinete architecture, host interaction, and midgut traversal, despite only modest alterations in overall protein abundance.

Table S1. Proteomics of *pk2ptd* versus WT GFP ookinetes 24 hpa.

Table S2. Phosphoproteomics of *pk2ptd* versus WT GFP ookinetes 24 hpa.

## Discussion

PKs are successfully targeted in the treatment of many diseases such as cancer (19), and efforts are being made to develop therapeutics that target *Plasmodium* kinases (13). PK2 function has been investigated in asexual blood-stage parasites, but little was known about its importance in transmission, so we investigated its role in the sexual stages of the life cycle. A previous study had begun to characterise *P. falciparum* PK2, showing that it was required for merozoite invasion (15). PK2 was found in distinct foci in schizonts; however, these did not colocalise well with components of the invasive machinery, such as micronemes or rhoptries. We have also found PK2 at discrete foci in *P. berghei* blood stages, not only in schizonts but also in rings, trophozoites, and merozoites.

Live-cell imaging of PK2-GFP parasites revealed that there is no detectable expression in male or female gametocytes. However, post-fertilisation, we found PK2 expression begins around ookinete development stage V, with an interesting pattern reminiscent of the contractile ring, similar to that of myosin F or kinesin-20 (20, 21). PK2 was present in mature ookinetes, diffusely distributed throughout the cytoplasm with some foci and a concentration at the apical and basal tip. PK2 was also expressed in sporozoites, displaying a distributed intracellular pattern with prominent internal/perinuclear signal and variable enrichment towards the apical tip, similar to the location of Myosin B and other conoid-associated proteins (22, 23, 24, 25). Together, these experiments showed that PK2 is expressed in all the invasive stages of the *P. berghei* life cycle, suggesting that it is involved as a common component of the invasion process in all three stages, not just in merozoites as described previously (15).

We show here that PK2 is required for the ookinete-to-oocyst transition in the mosquito midgut. The conditional knockdown approach that reduced PK2 transcript levels in the *pk2ptd* ookinete produced a mutant parasite unable to establish an infection in the mosquito midgut, potentially because of reduced motility of *pk2ptd* ookinetes. The significant reduction in *pk2ptd* ookinete motility likely compromises efficient traversal of the peritrophic matrix and mosquito midgut epithelium, thereby reducing the number of parasites reaching the basal surface where ookinete-to-oocyst differentiation occurs. Comparable phenotypes have been reported after disruption of CDPK3, CTRP, PPKL, and the alveolin IMC1b, all of which impair ookinete gliding or mechanical integrity and cause marked reductions in midgut invasion and oocyst formation (26, 27, 28, 29). Thus, impaired motility potentially contributes substantially to the near-complete loss of *pk2ptd* oocysts, although the failure of haemocoel-injected parasites to produce sporozoites indicates that PK2 also has an additional function after midgut traversal. Strikingly, even after direct injection of *pk2ptd* ookinetes into the mosquito haemocoel, the parasites were still unable to form oocysts and produce sporozoites. This further supports an additional requirement for PK2 after midgut traversal, although the precise developmental stage and function remain unclear. This phenotype is quite different to that of Shewanella-like protein phosphatase 1 mutants, which have a similar mislocalisation of micronemes but with a defect that was solely limited to ookinete invasion of the mosquito epithelial midgut (18). A similar phenotype was observed previously in a *Plasmodium* Infection of the Mosquito Midgut Screen 43 mutant: direct injection of these ookinetes into the haemocoel also failed to produce sporozoites (30).

The phosphoproteomic analysis provides some mechanistic insight into how PK2 may have key roles in transmission-stage development. Several proteins known to be essential for ookinete/oocyst development or sporogony were hypophosphorylated in *pk2ptd* ookinetes, in particular, ALV7 (also known as IMC1j), which has been shown to be essential for parasite fitness, subpellicular microtubule organisation, and ookinete motility (31); and the zinc finger protein, Pb103, vital for ookinete development (32). Collectively, these changes could support a model in which PK2 regulates a phosphorylation network spanning invasion-associated functions and developmental programmes required for ookinete-to-oocyst transition and parasite transmission. This profile differs from that resulting from the perturbation of PfPK2 in asexual blood stages, which was linked to cGMP/Ca^2+^-associated invasion signalling and changes in phosphorylation in invasion-related pathways (15), highlighting the context-specific functions of PK2-regulated networks in different life-cycle invasive stages. Indeed, only two proteins (PBANKA_1342300—conserved gene of unknown function, and PBANKA_1423500—60S ribosomal protein L13-2, putative) are present in the two datasets.

Our observation that *pk2ptd* mutant ookinetes had an altered location of micronemes is similar to that observed previously in a Shewanella-like protein phosphatase 1 knockout mutant (18). The release of microneme contents is necessary for ookinete invasion of the mosquito midgut (18, 33), and the secreted proteins have several functions in ookinete adhesion, motility, and membrane degradation. For example, CTRP is required for ookinete motility and its gene deletion results in a failure to establish midgut infection (29). CHT1 is required to degrade the peritrophic matrix of the gut, and its gene disruption significantly reduces midgut infection (34, 35). In contrast to other invasive stages of the life cycle, ookinetes lack both rhoptries and dense granules and invasion relies mainly on micronemes (36, 37).

Normally, micronemes bud off from the ER–Golgi secretory pathway and are transported along microtubules towards the apical end of the cell, eventually docking with rhoptry tips at the time of release (38, 39, 40). Mislocalisation of micronemes could indicate transport defects and may also indicate that they are either unable to secrete components required for invasion or are able to secrete but the components are in the wrong subcellular compartment, potentially explaining why so few oocysts are formed (18, 36). It is also possible that different types of microneme may exist (41). It will be important to determine whether different types of microneme exist in ookinete and sporozoites and whether they have functional differences. Our observations on the importance of micronemes fit with findings in *P. falciparum* merozoites, showing that secretion of microneme proteins is reduced in a conditional knockout mutant of *pk2* (41). Taken together, these findings suggest that PK2 has an important function with respect to micronemes, either in ensuring their correct positioning or directly or indirectly facilitating release of their contents in all three invasive stages of malaria parasite.

Overall, our data indicate that PK2 is a PK that is essential for ookinete-to-oocyst transition, thereby affecting parasite transmission. We propose that PK2 may regulate phosphorylation events associated with microneme distribution and ookinete-to-oocyst transition. These findings support the idea of PK2-regulated pathways as attractive candidates for transmission-blocking intervention strategies.

## Materials and Methods

### Ethics statement

Animal procedures were carried out in accordance with the Animal (Scientific Procedures) Act 1986 under the UK Home Office Licenses PDD2D5182; PP3589958. Female outbred CD1 mice aged 6–8 wk were used for all experiments. Mice were kept in a 12-h light, 12-h dark cycle at a temperature between 20 and 24°C with humidity between 40 and 60%.

### Generation of transgenic parasites

PbPK2-GFP was generated by amplifying a region of the *Pbpk2* gene downstream of the ATG start codon and ligating it to the p277 vector as described previously (33). The p277 vector contains the human *dhfr* cassette, conveying resistance to pyrimethamine. Schematic representations of the endogenous gene locus, the constructs, and the recombined gene locus can be found in Fig S1A. Diagnostic PCR was performed with primers IntT302 and ol492. For all primers used in the study, see Table S3.

Table S3. Primers used in the study.

The *pk2ptd* parasite line was generated using a conditional knockdown strategy, which places the *pk2* gene under the control of the *ama1* promoter as described previously (42). The knockdown construct was generated by double homologous recombination into the *Pama1* (pSS368) vector. A schematic representation of the endogenous *pk2* locus, the constructs, and the recombined *pk2* locus can be found in Fig S3A. Diagnostic PCR was performed for *pk2* gene knockdown parasites using primers IntPTD61_5 and 5′-IntPTD to determine successful integration of the targeting construct at the 5′ end of the gene locus. Primers IntPTD61_3 and 3′-IntAMA1 were used to determine successful integration for the 3′ end of the gene locus. Both constructs were transfected by electroporation (43).

### Purification of schizonts and gametocytes

Blood from infected mice (4 d post-infection) was placed in schizont medium (RPMI-1640 medium containing penicillin–streptomycin and heat-inactivated FBS) for 24 h at 37°C with shaking at 100 rpm. Schizonts were then purified on a 60% (in PBS) vol/vol Nycodenz column (Nycodenz stock solution: 27.6% wt/vol Nycodenz in 5 mM Tris–HCl [pH 7.20], 3 mM KCl, 0.3 mM EDTA). Schizonts were then harvested from the interface and used for downstream assays.

The purification of gametocytes was achieved by injecting parasites into phenylhydrazine-treated mice (44) and enriched by sulphadiazine treatment after 2 d of infection. The blood was collected on day 4 after infection, and gametocyte-infected cells were purified on a 48% vol/vol Nycodenz (in PBS) gradient (Nycodenz stock solution: 27.6% wt/vol Nycodenz in 5 mM Tris–HCl (pH 7.20), 3 mM KCl, 0.3 mM EDTA). The gametocytes were harvested from the interface and activated for 15 min to induce exflagellation.

### Ookinete culture and purification

Blood from infected mice was cultured in ookinete medium (RPMI-1640, 20% FBS, 100 μM xanthurenic acid) for up to 24 h to produce mature ookinetes. Ookinetes were purified using one of two methods: either by Nycodenz column (62.5% in PBS) when the aim of the experiment was RNA extraction or by magnetic beads coated in an antibody against surface protein P28, for U-ExM or TEM experiments.

### Live-cell imaging

To determine PK2 expression throughout the *Plasmodium* life cycle, small amounts of blood containing PK2-GFP parasites were mixed with PBS-DAPI and imaged to visualise blood-stage parasites. Blood was also placed into schizont culture and imaged at regular intervals over a period of 24 h. Blood was also added to ookinete media (RPMI media containing 100 μM xanthurenic acid, 1% wt/vol sodium bicarbonate, and 20% vol/vol heat-inactivated FBS) and ookinete development captured by live-cell imaging. 13.1-Cy3 antibody was used to identify the P28 surface protein expressed by zygotes and ookinete stages. Ookinete staging was based on criteria outlined in reference (9). Oocysts and sporozoites were imaged by removing the mosquito midguts/salivary glands from the mosquito. Salivary glands were homogenised in PBS-DAPI to visualise free sporozoites. For all imaging, either a 10× or 63× oil immersion objective was used to capture all images on a Zeiss Axio Imager M2 microscope fitted with an AxioCam ICc1 digital camera.

### Structured illumination microscopy

A small volume (3 μl) of parasite suspension (either in PBS: Hoechst for blood stages or in RPMI 1640 containing Hoechst dye and P28 antibody labelled with Cy3 for ookinetes) was placed on a microscope slide and covered with a long (50 × 34 mm) coverslip to obtain a very thin monolayer and immobilise the cells. Cells were scanned with an inverted microscope using a Zeiss Plan-Apochromat 63×/1.4 oil immersion or Zeiss C-Apochromat 63×/1.2 W Korr M27 water immersion objective on a Zeiss Elyra PS.1 microscope, using the SIM technique. The following settings were used in SIM mode: lasers, 405 nm: 40%, 488 nm: 17%, 561 nm: 20%; exposure times 109 ms (Hoechst), 122 ms (GFP for PK2), and 126 ms (dxRed for P28). The band-pass filters BP 420–480 + LP 750, BP 495–550 + LP 750, and BP 570–620 + 750 were used for the blue, green, and red channels, respectively.

### Parasite phenotype analysis

For analysis of male gametocyte exflagellation, mice were injected with blood containing ∼1 × 10^6^ parasites. 4–5 d later, blood was collected and exflagellation centres were counted by activating a small volume of blood in ookinete media for 15 min. For ookinetes, parasite-infected blood was incubated in ookinete media at 20°C for 24 h and ookinete conversion was quantified using 13.1 antibody. To assess oocyst formation and development, 30–50 *A*. *stephensi* SD 500 mosquitoes were allowed to feed for 20 min on anaesthetised, infected mice that had comparable parasitaemia and gametocytaemia. Around 10 midguts were removed from infected mosquitoes on day 7, 14, and 21 post-feeding and imaged to assess oocyst number. Mosquito bite-back experiments were performed by allowing infected mosquitoes to feed on anaesthetised, uninfected mice. Blood smears were made from these mice and days counted until the mice became positive for parasites.

### Ookinete injection experiments

3- to 5-d-old female *A. stephensi* mosquitoes were infected by microinjection of 69 nl of in vitro cultured ookinetes into the thorax using glass capillary needles and the Nanoject II microinjector (Drummond Scientific). Briefly, WT GFP and *pk2ptd* ookinetes were cultured as described. Blood from infected mice was collected via cardiac puncture and placed in 30 volumes of the ookinete medium (RPMI 1640 supplemented with 25 mM Hepes, 0.4 mM hypoxanthine, 0.1 mM xanthurenic acid, 24 mM of NaHCO_3_, and 100 U/ml of penicillin/streptomycin solution [Thermo Fisher Scientific]) before incubation for 24 h at 21°C. Ookinete numbers from 22- to 24-h in vitro cultures were counted using a standard haemocytometer, and ookinete density was adjusted to 1.2 or 2.4 × 10^4^ per μl for a final concentration of ∼800 or 1,600 ookinetes per mosquito, respectively. At 20–21 dpbi, 25 salivary glands were dissected and homogenised in PBS, and salivary gland sporozoites were counted using a standard haemocytometer.

### Western blotting

Purified schizonts were lysed in lysis buffer (10 mM Tris–HCl [pH 7.5], 150 mM NaCl, 0.5 mM EDTA, and 1% NP-40). Lysates were then mixed with Laemmli buffer and boiled at 95°C for 10 min before being centrifuged at 2,400*g* for 5 min. Samples were run on a 4–12% SDS–PAGE gel and transferred to nitrocellulose membrane (Amersham Biosciences). Immunoblotting was performed using a WesternBreeze Chemiluminescence anti-rabbit kit (Invitrogen) according to the manufacturer’s instructions. Primary antibody used was anti-GFP polyclonal antibody (Invitrogen) (1:1,250).

### U-ExM and microneme quantification

Fixed cells were attached to 12-mm poly-D-lysine coverslips (A3890401; Gibco) for 30 min. Coverslips were then incubated at 4°C overnight in 1.4% formaldehyde and 2% acrylamide in PBS. The next day, gels were prepared using monomer solution (23% sodium acrylate, 10% acrylamide, 0.1% bis-acrylamide in PBS) and by setting the monomer solution using 10% APS and 10% TEMED. Gels were incubated for 30 min at 37°C. Gels were then denatured for 15 min at 37°C and then transferred to Eppendorf tubes and boiled at 95°C for 45 min. Gels were then left in distilled water overnight to expand. The next day, gels were washed in PBS for 15 min before being incubated in primary antibodies diluted in 3% BSA at 4°C overnight. Gels were then washed three times in 0.1% PBS/Tween, 10 min per wash. Gels were then incubated in secondary antibodies for 3 h at room temperature before being washed in 0.1% PBST as described. Gels were again left to expand in distilled water overnight (method adapted from Liffner et al (38)).

Primary antibodies used were α-tubulin (T9026; Sigma-Aldrich, 1:1,000) and GFP (Thermo Fisher Scientific, 1:250). Secondary antibodies used were mouse Alexa Fluor 488 (1:1,000) and rabbit Alexa Fluor 568 (1:1,000). Stains used were Atto 594 NHS-ester (08741; Merck, 1:200). Gels were imaged in glass-bottom dishes (P35GC-1.5-14-C; MatTek). A Zeiss Elyra PS.1/LSM 780 microscope was used to image gels. Image analysis was performed in Zeiss Zen 2012 Black edition or ImageJ.

For quantification of apical-end micronemes, microneme-like structures were identified as discrete NHS-ester–positive puncta within the parasite cytoplasm. Structures were counted from maximum-intensity projections. The apical region was defined as the anterior one-third of the ookinete, identified from its tapered morphology and the organisation of the subpellicular microtubules. Images were acquired using identical settings.

### Electron microscopy

Ookinetes were purified using 13.1-coated beads and then fixed in 4% glutaraldehyde in 0.1 M phosphate buffer and processed for electron microscopy. Briefly, samples were post-fixed in 1% osmium tetroxide in 1.5% potassium ferrocyanide in 0.1 M phosphate buffer for 2 h at room temperature in the dark. Samples were then incubated in 2% uranyl acetate in ddH_2_O for 90 min, dehydrated in ethanol (a progressive series of ethanol concentrations from 20%, 40%, 90%, to 100% with two changes in absolute ethanol), and embedded in TAAB 812 Hard resin (TAAB, catalogue number T030). Ultrathin sections (∼70 nm) were cut from the sample using a diamond knife and mounted on copper grids. The grids were post-stained with 5% uranyl acetate followed by lead citrate to enhance contrast. Sections were examined using a JEOL JEM 1400 Flash transmission electron microscope (JEOL) at 120 kV.

### RNA isolation and qRT–PCR

RNA was extracted from purified schizonts and ookinetes using an RNA extraction kit (Stratagene) according to the manufacturer’s instructions. Complementary DNA was made using a High-Capacity RNA-to-cDNA kit (Applied Biosystems) according to the manufacturer’s instructions. Gene expression was quantified from 80 ng total cDNA using SYBR Green Fast Master Mix Kit (Applied Biosystems). Primers were designed using Primer3 (Primer-BLAST, NCBI). A 7500 FAST machine (Applied Biosystems) was used to perform the analysis with the following cycling conditions: 95°C for 20 s, followed by 40 cycles of 95°C for 3 s and 60°C for 30 s. Two technical replicates and three biological replicates were performed for each gene tested. Two control reference genes were used: *arginyl-tRNA synthetase* (PBANKA_1434200) and *hsp70* (PBANKA_0818900). Primers used are listed in Table S3.

### Ookinete motility assay and quantification

Our ookinete motility assay was previously described in Zeeshan et al (20). Briefly, blood was taken from mice and incubated in double the volume of ookinete media for 24 h. Cells were resuspended and mixed with Matrigel (Corning) in a 1:1 ratio. Cells were then added to a microscope slide, a coverslip was placed over them, and nail polish was used to seal the coverslip to the slide. Slides were allowed to set for 30 min before imaging. Time series images were captured on the Zeiss Axio Imager microscope previously described, using the 63× objective and DIC imaging. Analysis was performed in ImageJ using the Manual Tracking plug-in. Briefly, a spot was placed at the apical end of the ookinete in all images in a time series. Manual tracking allowed the path taken by the spot to be plotted and measured giving measurements for distance and speed. Distances between frames were added together to give total distance travelled. Measurements for speed were averaged to give mean speed travelled.

### Phosphoproteomics

#### Sample preparation (SDS buffer-FASP procedure)

Ookinetes of WT GFP and *pk2ptd* lines at 24 hpa (each from three biological replicates) were lysed in 400 μl of 2% SDS, 25 mM NaCl, 50 mM Tris (pH 7.4), 2.5 mM EDTA, and 20 mM TCEP supplemented with 1x Halt protease and phosphatase inhibitor. Samples were vortexed and then heated at 95°C for 10 min with shaking at 400 rpm on a thermomixer. DNA was sheared via four sonication pulses of 10 s each at 50% power. Samples were then centrifuged for 30 min at 17,000*g*, and the supernatant was collected. After protein assay, a SDS–PAGE was performed to assess the quality of the samples and the volume corresponding to 600 μg of protein was taken for each sample, completed to 300 μl with Tris–HCl 0.1M, pH 8.5, and the samples were incubated with 48 μl of iodoacetamide 0.5 M for 1 h at room temperature. Proteins were digested based on the FASP method (45) using Amicon Ultra-4 30 kD as centrifugal filter units (Millipore). Trypsin (Promega) was added at 1:80 enzyme/protein ratio, and digestion was performed overnight at room temperature. The resulting peptide samples were desalted using Pierce Peptide Desalting Spin Columns (Thermo Fisher Scientific) according to the manufacturer’s instruction and then completely dried under speed-vacuum.

#### TMT11plex-labelling procedure

Peptide concentration was determined using a colorimetric peptide assay (Thermo Fisher Scientific). Briefly, 140 μg of each sample was labelled with 800 μg of the corresponding TMT11plex reagent previously dissolved in 220 μl of 36% CH_3_CN, 200 mM EPPS (pH 8.5). The reaction was performed for 1 h at room temperature and then quenched by adding hydroxylamine to a final concentration of 0.3% (vol/vol). Labelled samples were pooled together, dried under speed-vacuum, and desalted using Pierce Peptide Desalting Spin Columns (Thermo Fisher Scientific) according to the manufacturer’s instruction, and then completely dried under speed-vacuum. The tagging efficiency was around 90%.

#### Phosphopeptide enrichment

Phosphopeptides were enriched using High-Select Fe-NTA Phosphopeptide Enrichment Kit (Thermo Fisher Scientific) following the manufacturer’s instructions. Phosphopeptide fraction and flow-through fraction were desalted with Pierce Peptide Desalting Spin Columns (Thermo Fisher Scientific) according to the manufacturer’s instruction and then completely dried under speed-vacuum.

#### ESI-LC-MS/MS

Samples were reconstituted in loading buffer (5% CH3CN, 0.1% FA), and the peptide concentration was determined using a colorimetric peptide assay (Thermo Fisher Scientific). For the flow-through and for the phosphopeptide-enriched fractions, 3 μg was injected on a column. LC-ESI-MS/MS was performed on an Orbitrap Fusion Lumos Tribrid mass spectrometer (Thermo Fisher Scientific) equipped with a Vanquish NEO liquid chromatography system (Thermo Fisher Scientific). Peptides were trapped on an Acclaim PepMap100, C18, 3 μm, 75 μm × 20 mm nanotrap column (Thermo Fisher Scientific) and separated on a 75 μm × 500 mm, C18 ReproSil-Pur (Dr. Maisch GmBH), 1.9 μm, 100 Å, home-made column. The analytical separation was run for 180 min using a gradient of H_2_O/FA 99.9%/0.1% (solvent A) and CH_3_CN/H_2_O/FA 80.0%/19.9%/0.1% (solvent B). The gradient was run from 5% B to 28% B in 160 min, then to 40% B in 20 min, then to 99% B in 10 min with a final stay of 20 min at 99% B. The flow rate was of 250 nl/min, and the total run time was of 210 min. Data-Dependent Acquisition was performed with MS1 full scan at a resolution of 120,000 FWHM followed by as many subsequent MS2 scans on selected precursors as possible within 3-s maximum cycle time. MS1 was performed in the Orbitrap with an AGC target of 4 × 10^5^, a maximum injection time of 50 ms, and a scan range from 375 to 1,500 m/z. MS2 was performed in the Orbitrap at a resolution of 50,000 FWHM using higher energy collisional dissociation at 38% NCE. Isolation window was at 0.7 μ with an AGC target of 5 × 104 and a maximum injection time of 86 ms. A dynamic exclusion of parent ions of 60 s with 10 ppm mass tolerance was applied.

#### Database search

Raw data were processed using Proteome Discoverer 2.4 software (Thermo Fisher Scientific). Briefly, spectra were extracted and searched against the *P. berghei* ANKA database (PlasmoDB.org, release 68, 4,958 entries), the *Mus musculus* reference proteome database (UniProt, reviewed, release 2025_08, 17,234 entries), and an in-house database of common contaminants using Mascot (Matrix Science; version 2.6.2). Trypsin was selected as the enzyme, with one potential missed cleavage. Precursor ion tolerance was set to 20 ppm and fragment ion tolerance to 0.02 D. Carbamidomethylation of cysteine (+57.021), TMT6plex (+229.163) on lysine residues, and TMT6plex (+229.163) on peptide N-termini were specified as fixed modification. Oxidation of methionine (+15.995) was set as variable modifications, as well as phosphorylation of serine, threonine, and tyrosine. The search results were validated with Percolator. PSM and peptides were filtered with a false discovery rate of 1%, and then grouped to proteins with again a false discovery rate of 1% and using only peptides with high confidence level. Both unique and razor peptides were used for quantitation, and protein and peptide abundance values were based on S/N values of reporter ions. The abundance was normalised on “Total Peptide Amount” excluding phosphopeptides and then scaled with the “On all Average” parameter. The protein ratios were directly calculated from the grouped protein abundance, and associated *P*-values were calculated with ANOVA test based on the abundance of individual proteins or peptides. The ptmRS mode was used for phosphorylation modification site localisation and validation. Only phosphosites with a probability of more than 75% are considered as validated.

### Statistical analysis

All graphs and statistical analysis were produced in GraphPad Prism 10. *t* tests were used to assess differences between control and experimental groups. Statistical significance is denoted as *P < 0.05, **P < 0.01, ***P < 0.001, ****P < 0.0001, or ns = non-significant.

## Supplementary Material

Reviewer comments

## Data Availability

The mass spectrometry proteomics data have been deposited to the ProteomeXchange Consortium via the PRIDE84 partner repository with the dataset identifier PXD081992 (proteomics and phosphoproteomics). The authors declare that all other relevant data generated or analysed during this study are included in the article or its supplementary information. Raw data are available for each figure. Materials are available from the corresponding authors on reasonable request. All other source data are provided with this study.

## References

[1] Poespoprodjo JR, Douglas NM, Ansong D, Kho S, Anstey NM (2023) Malaria. Lancet 402: 2328–2345. 10.1016/S0140-6736(23)01249-7 37924827

[2] Adderley J, Williamson T, Doerig C (2021) Parasite and host erythrocyte kinomics of *Plasmodium* infection. Trends Parasitol 37: 508–524. 10.1016/j.pt.2021.01.002 33593681

[3] Guttery DS, Zeeshan M, Ferguson DJP, Holder AA, Tewari R (2022) Division and transmission: Malaria parasite development in the mosquito. Annu Rev Microbiol 76: 113–134. 10.1146/annurev-micro-041320-010046 35609946

[4] Guttery DS, Zeeshan M, Holder AA, Tewari R (2024) The molecular mechanisms driving *Plasmodium* cell division. Biochem Soc Trans 52: 593–602. 10.1042/BST20230403 PMC11088906 38563493

[5] Guttery DS, Zeeshan M, Holder AA, Tromer EC, Tewari R (2023) Meiosis in *Plasmodium*: How does it work? Trends Parasitol 39: 812–821. 10.1016/j.pt.2023.07.002 37541799

[6] Roques M, Bindschedler A, Beyeler R, Heussler VT (2023) Same, same but different: Exploring *Plasmodium* cell division during liver stage development. PLoS Pathog 19: e1011210. 10.1371/journal.ppat.1011210 PMC10062574 36996035

[7] Kanatani S, Stiffler D, Bousema T, Yenokyan G, Sinnis P (2024) Revisiting the *Plasmodium* sporozoite inoculum and elucidating the efficiency with which malaria parasites progress through the mosquito. Nat Commun 15: 748. 10.1038/s41467-024-44962-4 PMC10811227 38272943

[8] Molina-Franky J, Patarroyo ME, Kalkum M, Patarroyo MA (2022) The cellular and molecular interaction between erythrocytes and *Plasmodium falciparum* merozoites. Front Cell Infect Microbiol 12: 816574. 10.3389/fcimb.2022.816574 PMC9008539 35433504

[9] Janse CJ, Mons B, Rouwenhorst RJ, Van der Klooster PF, Overdulve JP, Van der Kaay HJ (1985) In vitro formation of ookinetes and functional maturity of *Plasmodium berghei* gametocytes. Parasitology 91: 19–29. 10.1017/s0031182000056481 2863802

[10] Smith RC, Barillas-Mury C (2016) *Plasmodium* oocysts: Overlooked targets of mosquito immunity. Trends Parasitol 32: 979–990. 10.1016/j.pt.2016.08.012 27639778

[11] Tewari R, Straschil U, Bateman A, Bohme U, Cherevach I, Gong P, Pain A, Billker O (2010) The systematic functional analysis of *Plasmodium* protein kinases identifies essential regulators of mosquito transmission. Cell Host Microbe 8: 377–387. 10.1016/j.chom.2010.09.006 PMC2977076 20951971

[12] Ward P, Equinet L, Packer J, Doerig C (2004) Protein kinases of the human malaria parasite *Plasmodium falciparum*: The kinome of a divergent eukaryote. BMC Genomics 5: 79. 10.1186/1471-2164-5-79 PMC526369 15479470

[13] Arendse LB, Wyllie S, Chibale K, Gilbert IH (2021) *Plasmodium* kinases as potential drug targets for malaria: Challenges and opportunities. ACS Infect Dis 7: 518–534. 10.1021/acsinfecdis.0c00724 PMC7961706 33590753

[14] Solyakov L, Halbert J, Alam MM, Semblat JP, Dorin-Semblat D, Reininger L, Bottrill AR, Mistry S, Abdi A, Fennell C, (2011) Global kinomic and phospho-proteomic analyses of the human malaria parasite *Plasmodium falciparum*. Nat Commun 2: 565. 10.1038/ncomms1558 22127061

[15] Rawat RS, Gupta A, Antil N, Bhatnagar S, Singh M, Rawat A, Prasad TSK, Sharma P (2023) Protein kinase PfPK2 mediated signalling is critical for host erythrocyte invasion by malaria parasite. PLoS Pathog 19: e1011770. 10.1371/journal.ppat.1011770 PMC10662742 37988347

[16] Kato K, Sudo A, Kobayashi K, Tohya Y, Akashi H (2008) Characterization of *Plasmodium falciparum* protein kinase 2. Mol Biochem Parasitol 162: 87–95. 10.1016/j.molbiopara.2008.07.007 18762219

[17] Sebastian S, Brochet M, Collins MO, Schwach F, Jones ML, Goulding D, Rayner JC, Choudhary JS, Billker O (2012) A *Plasmodium* calcium-dependent protein kinase controls zygote development and transmission by translationally activating repressed mRNAs. Cell Host Microbe 12: 9–19. 10.1016/j.chom.2012.05.014 PMC3414820 22817984

[18] Patzewitz EM, Guttery DS, Poulin B, Ramakrishnan C, Ferguson DJ, Wall RJ, Brady D, Holder AA, Szöőr B, Tewari R (2013) An ancient protein phosphatase, SHLP1, is critical to microneme development in *Plasmodium* ookinetes and parasite transmission. Cell Rep 3: 622–629. 10.1016/j.celrep.2013.01.032 PMC3617505 23434509

[19] Riegel K, Vijayarangakannan P, Kechagioglou P, Bogucka K, Rajalingam K (2022) Recent advances in targeting protein kinases and pseudokinases in cancer biology. Front Cell Dev Biol 10: 942500. 10.3389/fcell.2022.942500 PMC9354965 35938171

[20] Zeeshan M, Rashpa R, Ferguson DJP, Abel S, Chahine Z, Brady D, Vaughan S, Moores CA, Le Roch KG, Brochet M, (2022) Genome-wide functional analysis reveals key roles for kinesins in the mammalian and mosquito stages of the malaria parasite life cycle. PLoS Biol 20: e3001704. 10.1371/journal.pbio.3001704 PMC9333250 35900985

[21] Wall RJ, Zeeshan M, Katris NJ, Limenitakis R, Rea E, Stock J, Brady D, Waller RF, Holder AA, Tewari R (2019) Systematic analysis of *Plasmodium* myosins reveals differential expression, localisation, and function in invasive and proliferative parasite stages. Cell Microbiol 21: e13082. 10.1111/cmi.13082 PMC6851706 31283102

[22] Kwecka D, Wang Z, Eigminas E, Liu L, Regan JC, Kim C, Philip N (2025) A divergent cyclic nucleotide binding protein promotes *Plasmodium* ookinete infection of the mosquito. PLoS Pathog 21: e1013467. 10.1371/journal.ppat.1013467 PMC12422582 40892844

[23] Bertiaux E, Balestra AC, Bournonville L, Louvel V, Maco B, Soldati-Favre D, Brochet M, Guichard P, Hamel V (2021) Expansion microscopy provides new insights into the cytoskeleton of malaria parasites including the conservation of a conoid. PLoS Biol 19: e3001020. 10.1371/journal.pbio.3001020 PMC7951857 33705377

[24] Wall RJ, Roques M, Katris NJ, Koreny L, Stanway RR, Brady D, Waller RF, Tewari R (2016) SAS6-like protein in *Plasmodium* indicates that conoid-associated apical complex proteins persist in invasive stages within the mosquito vector. Sci Rep 6: 28604. 10.1038/srep28604 PMC4919640 27339728

[25] Yusuf NA, Green JL, Wall RJ, Knuepfer E, Moon RW, Schulte-Huxel C, Stanway RR, Martin SR, Howell SA, Douse CH, (2015) The *Plasmodium* class XIV Myosin, MyoB, has a distinct subcellular location in invasive and motile stages of the malaria parasite and an unusual light chain. J Biol Chem 290: 12147–12164. 10.1074/jbc.M115.637694 PMC4424349 25802338

[26] Siden-Kiamos I, Ecker A, Nyback S, Louis C, Sinden RE, Billker O (2006) *Plasmodium berghei* calcium-dependent protein kinase 3 is required for ookinete gliding motility and mosquito midgut invasion. Mol Microbiol 60: 1355–1363. 10.1111/j.1365-2958.2006.05189.x PMC1513514 16796674

[27] Tremp AZ, Khater EI, Dessens JT (2008) IMC1b is a putative membrane skeleton protein involved in cell shape, mechanical strength, motility, and infectivity of malaria ookinetes. J Biol Chem 283: 27604–27611. 10.1074/jbc.M801302200 PMC2562075 18650444

[28] Guttery DS, Poulin B, Ferguson DJ, Szoor B, Wickstead B, Carroll PL, Ramakrishnan C, Brady D, Patzewitz EM, Straschil U, (2012) A unique protein phosphatase with kelch-like domains (PPKL) in *Plasmodium* modulates ookinete differentiation, motility and invasion. PLoS Pathog 8: e1002948. 10.1371/journal.ppat.1002948 PMC3447748 23028336

[29] Dessens JT, Beetsma AL, Dimopoulos G, Wengelnik K, Crisanti A, Kafatos FC, Sinden RE (1999) CTRP is essential for mosquito infection by malaria ookinetes. EMBO J 18: 6221–6227. 10.1093/emboj/18.22.6221 PMC1171685 10562534

[30] Ukegbu CV, Giorgalli M, Tapanelli S, Rona LDP, Jaye A, Wyer C, Angrisano F, Blagborough AM, Christophides GK, Vlachou D (2020) PIMMS43 is required for malaria parasite immune evasion and sporogonic development in the mosquito vector. Proc Natl Acad Sci U S A 117: 7363–7373. 10.1073/pnas.1919709117 PMC7132314 32165544

[31] He G, Feng Y, He D, Hu Y, Wang D, Wang S, Sun X, Shi S, Yang T, Hu X, (2025) *Plasmodium berghei* IMC1j interacts with δ-tubulin to orchestrate subpellicular microtubule organization in ookinetes. mBio 16: e0266925. 10.1128/mbio.02669-25 PMC12607714 41117539

[32] Hirai M, Maeta A, Mori T, Mita T (2021) Pb103 regulates zygote/ookinete development in *Plasmodium berghei* via double zinc finger domains. Pathogens 10: 1536. 10.3390/pathogens10121536 PMC8707419 34959491

[33] Guttery DS, Poulin B, Ramaprasad A, Wall RJ, Ferguson DJ, Brady D, Patzewitz EM, Whipple S, Straschil U, Wright MH, (2014) Genome-wide functional analysis of *Plasmodium* protein phosphatases reveals key regulators of parasite development and differentiation. Cell Host Microbe 16: 128–140. 10.1016/j.chom.2014.05.020 PMC4094981 25011111

[34] Tsai YL, Hayward RE, Langer RC, Fidock DA, Vinetz JM (2001) Disruption of *Plasmodium falciparum* chitinase markedly impairs parasite invasion of mosquito midgut. Infect Immun 69: 4048–4054. 10.1128/IAI.69.6.4048-4054.2001 PMC98468 11349075

[35] Dessens JT, Mendoza J, Claudianos C, Vinetz JM, Khater E, Hassard S, Ranawaka GR, Sinden RE (2001) Knockout of the rodent malaria parasite chitinase pbCHT1 reduces infectivity to mosquitoes. Infect Immun 69: 4041–4047. 10.1128/IAI.69.6.4041-4047.2001 PMC98467 11349074

[36] Ukegbu CV, Gomes AR, Giorgalli M, Campos M, Bailey AJ, Besson TRB, Billker O, Vlachou D, Christophides GK (2023) Identification of genes required for *Plasmodium* gametocyte-to-sporozoite development in the mosquito vector. Cell Host Microbe 31: 1539–1551.e6. 10.1016/j.chom.2023.08.010 PMC7618085 37708854

[37] Lal K, Prieto JH, Bromley E, Sanderson SJ, Yates JR, Wastling JM, Tomley FM, Sinden RE (2009) Characterisation of *Plasmodium* invasive organelles; an ookinete microneme proteome. Proteomics 9: 1142–1151. 10.1002/pmic.200800404 PMC2706521 19206106

[38] Liffner B, Cepeda Diaz AK, Blauwkamp J, Anaguano D, Frolich S, Muralidharan V, Wilson DW, Dvorin JD, Absalon S (2023) Atlas of *Plasmodium falciparum* intraerythrocytic development using expansion microscopy. Elife 12: RP88088. 10.7554/eLife.88088 PMC10727503 38108809

[39] Kats LM, Cooke BM, Coppel RL, Black CG (2008) Protein trafficking to apical organelles of malaria parasites - building an invasion machine. Traffic 9: 176–186. 10.1111/j.1600-0854.2007.00681.x 18047549

[40] Bannister LH, Hopkins JM, Dluzewski AR, Margos G, Williams IT, Blackman MJ, Kocken CH, Thomas AW, Mitchell GH (2003) *Plasmodium falciparum* apical membrane antigen 1 (PfAMA-1) is translocated within micronemes along subpellicular microtubules during merozoite development. J Cell Sci 116: 3825–3834. 10.1242/jcs.00665 12902400

[41] Darif N, Rheinnecker M, Hildenbrand K, Chookajorn T, Dorner LP, Heriche JK, Henriksson S, Funaya C, Hentzschel F, Sandblad L, (2026) Cellular hallmarks from volume electron microscopy reveal developmental progression of *Plasmodium* ookinetes. Adv Sci (Weinh) 13: e08250. 10.1002/advs.202508250 PMC12822446 41025598

[42] Nagar A, Yanase R, Zeeshan M, Ferguson DJP, Abel S, Pashley SL, Mishra A, Eze A, Rea E, Brady D, (2026) *Plasmodium* ARK1 regulates spindle formation during atypical mitosis and forms a divergent chromosomal passenger complex. Nat Commun 17: 1598. 10.1038/s41467-026-69460-7 PMC12946202 41748607

[43] Janse CJ, Ramesar J, Waters AP (2006) High-efficiency transfection and drug selection of genetically transformed blood stages of the rodent malaria parasite *Plasmodium berghei*. Nat Protoc 1: 346–356. 10.1038/nprot.2006.53 17406255

[44] Beetsma AL, van de Wiel TJ, Sauerwein RW, Eling WM (1998) *Plasmodium berghei* ANKA: Purification of large numbers of infectious gametocytes. Exp Parasitol 88: 69–72. 10.1006/expr.1998.4203 9501851

[45] Wisniewski JR, Zougman A, Nagaraj N, Mann M (2009) Universal sample preparation method for proteome analysis. Nat Methods 6: 359–362. 10.1038/nmeth.1322 19377485

