# Supplementary material for: Plasmodium protein kinase 2 is required for ookinete-to-oocyst transition and parasite transmission
Source: Life Sci Alliance. 2026 Oct 5;9(12):e202603863. doi: 10.26508/lsa.202603863 (PMC13646729; doi:10.26508/lsa.202603863)
Supplement: Supplementary file 1 [file LSA-2026-03863_review_history.pdf]

# Plasmodium Protein Kinase 2 is required for ookinete to oocyst transition and parasite transmission

Sarah Pashley, Molly Hair, Chiamaka Ukegbu, Mohammad Zeeshan, Akancha Mishra, Declan Brady, Robert Markus, Sue Vaughan, Carla Pasquarello, Anthony Holder, Alexandre Hainard, George Christophides, Dina Vlachou, Pushkar Sharma, David Guttery, and Rita Tewari
**DOI: https://doi.org//10.26508/lsa.202603863**

*Corresponding author(s): Rita Tewari, University of Nottingham*



## Review Timeline:

| | |
|---|---|
| Submission Date: | 2026-07-29 |
| Editorial Decision: | 2026-09-11 |
| Revision Received: | 2026-09-21 |
| Accepted: | 2026-09-21 |

*Scientific Editor: Tim Fessenden*


## Transaction Report:

(Note: With the exception of the correction of typographical or spelling errors that could be a source of ambiguity, letters and reports are not edited. The original formatting of letters and referee reports may not be reflected in this compilation.)

Please note that the manuscript was reviewed at *Review Commons* and these reports were taken into account in the decision-making process at *Life Science Alliance.*

Reviews

**Review #1**

**1. Evidence, reproducibility and clarity:**

**Summary**

The authors here characterize the function of the protein kinase PK2 in Plasmodium berghei mosquito stages. They find that besides in asexual blood stages, PK2 is expressed in mature ookinetes, oocysts and sporozoites, with a putative localisation at the apical tip of the invasive stages. Conditional depletion of PK2 in mosquito-infecting stages results in slower ookinetes and an almost complete abolishment of oocyst formation which cannot be rescued by injecting ookinetes into the haemocoel, thus bypassing the midgut invasion step. Expansion and electron microscopy reveals a reduced number of micronemes in ookinetes, possibly explaining the gliding and invasion effect. The authors perform phosphoproteomics to determine targets of PK2 in ookinetes. PK2 is a major kinase of Plasmodium parasites and the characterisation of its role in mosquito stages is interesting. Yet the localisation of PK2 does currently not convincingly support an apical localisation. It is clear that PK2 is important for mosquito infection, however, its precise phenotype could be defined a bit more, and the phosphoproteomics are inconclusive with regards to the mechanism of action. Also, the data presentation should be improved to support their statements and to be transparent about the number of replicates in each experiment. Specific major and minor points of critique are indicated below.

**Major points**

1) Putative apical localisation of PK2

At the end of the second result section, the authors summarize their findings with "Collectively, these GFP fluorescence data indicate that PK2-GFP is predominantly located at the apical end of all invasive stages". Yet, this statement is not supported by the presented data: In all presented stages, the majority of the GFP signal is found in the cytoplasm or partially in the nucleus, with different levels of speckles. There is no apical localisation visible in schizonts. While there might be an apical punctuate signal in some ookinetes, the images and their quantification are not yet convincing, and "predominantly apical localisation" does not hold true. This is especially true for the expansion microscopy images, where the GFP signal looks very noisy. The authors should present more images than the single example image they currently show for each stage and microscopy technique to support their statements about localisation. They should also state clear criteria how they define and quantify "apical localisation" and provide absolute numbers how many images were taken and analysed per stage in addition to percentages. One way to quantify would be to draw lines across either the apical tip or randomly selected lateral points of their target cells and plot GFP intensity profiles (e.g. in relation to MyoA-mCherry). Imaging with less background (e.g. by generating a new cell line, see the next point) and providing images of wildtype parasites stained with the same conditions (especially for Expansion Microscopy) to control for unspecific antibody binding and noise could also help to provide more convincing localisation data.

Importantly, the Western blot presented in Suppl Fig 1C suggests a considerable amount of cleaved GFP, possibly confounding the localisation and partially explaining the pan-cellular distribution of the signal. The authors should comment in their results that the localisation could be partially confounded by cleaved GFP. A better, albeit more work intensive solution would be to generate a new parasite line testing either a different linker between the GFP and PK2 or using an epitope tag, e.g. FLAG or HA, combined with immunofluorescence assays, to test the localisation of PK2. FLAG/HA-Tags might also help to reduce the speckled background in expansion microscopy.

If new experiments and data analysis do not show convincingly a consistent PK2-GFP localisation at the apical tip of all stages, the authors should tone down their conclusion. PK2 may well have a function in microneme formation/secretion without being localised at the apical tip.

Fig 2B: The authors write "The PK2-GFP signal showed limited overlap with that of the intranuclear spindle microtubules, indicating that PK2 is extra-nuclear and not a major component of the mitotic spindle apparatus in schizonts (Fig. 2B)." How are the authors sure that these are intranuclear spindles? To me these tubulin structures do not look like spindles, but like the subpellicular microtubules surrounding the forming merozoites (in line with the non-nuclear localisation of these tubulin structures e.g. in the lower right merozoite).

2) PK2 knockdown phenotype

While ookinetes seem to be a bit slower, this phenotype is not sufficient to explain the total absence of oocysts, as also other slow-moving ookinetes successfully invade mosquito midguts, at least to some extend (see e.g. PMID: 27084458, 35506479). Thus, PK2 likely has another phenotype either for ookinete-to-oocyst transition or for oocyst growth. Indeed, when the authors bypass the midgut traversal step, PK2-knockdown parasites still do not form sporozoites, indicating that midgut traversal is not the (only) limiting factor of the PK2 knockdown. Do the parasites form oocysts after hemocoel injection, and do these grow and sporulate? This would be important to conclude if PK2 is already essential for ookinete-to-oocyst conversion, if it is important for oocyst growth or instead only for sporozoite formation. Right now the data seems to points towards ookinete-to-oocyst transition, which would not be invasion and thus not be related to secretory vesicles.

Once they have pinpointed the point of arrest of the knockout better, the authors could further streamline the discussion and using more precise language depending on the actual phenotype (for example, the authors currently state that PK2 is essential for oocyst viability, and they also state that PK2 has a role in sporozoite development, when so far the data only shows that PK2 is essential for oocyst formation with some evidence that it is important for ookinete motility and microneme formation).

The proteome and phosphoproteome are unfortunately not very informative regarding the mechanism of action of PK2. Do the authors have an explanation why the (I assume) asexual protein EMAP1 is such a significant hit for hypophosphorylation? Is this a target of PK2 in blood stages? Generally, how much does the phosphoproteome of PK2-PTD in ookinetes overlap with the published blood-stage phosphoproteome of PK2 depletion? One would assume that at least some of the targets are conserved. How did the authors purify ookinetes and could it be that residual asexual parasites in the culture (which still express PK2) confound the data? Could that explain why PK2 does not appear in the proteome as down-regulated? Without robust downregulation of the targeted protein, it is difficult to draw conclusions about other deregulated proteins. The authors should also state somewhere how many replicates were done for the proteome and the phosphoproteome.

3) Data presentation and statistics
Using a bar graph and SEM is not appropriate for data with only two data points/biological replicates (e.g. Fig 3E, I, Suppl Fig. 2C-F). Showing individual values (as scatter plot) would be more appropriate. Also, how did the authors do statistics on only two replicates in Suppl fig. 2C, D? Were indeed technical and biological replicates pooled to obtain n=4 as the text in the statistical analysis section seems to suggest? This would not be correct as the error between technical replicates is much smaller that the variation between biological replicates. To perform statistical analysis, at least 3, better more, biological replicates (each the mean of appropriate number of technical replicates) are required. Alternatively, the authors can just depict the individual results of the biological replicates as a scatter plot and leave it to the reader to judge if this is a clear deregulation of expression. Please also indicate to what the expression is normalised. I would recommend scatter plots also for all experiments with only 3 data points (e.g. 3B, H). For Fig 3E, does independent experiments mean independent mosquito cages or just two mosquitoes?

**Minor points**

Ookinete stages: There is no consensus in the field about how many distinct "stages" an ookinete undergoes during maturation (it is likely a continuous process, anyways). While Siciliano et al (PMID 32256458) define five stages and Darif et al (PMID 41025598) define seven stages, the authors divide maturation here into six stages. Which criteria did the authors use to define the stages in their images? If the ookinete staging here really is important for localisation and characterisation of PK2 (one could argue it is not), the authors should include a scheme and example images in the supplements, otherwise just forgo any staging and use the wording early and late retorts with respective example images.

Fig 1B: The authors refer to Fig 1B for various statements regarding PK2-GFP expression and localisation, e.g.: "No detectable GFP signal was observed [...] in zygotes during early ookinete differentiation (stages I-III) (Fig. 1B)", "PK2-GFP was visible in stage IV ookinetes ", "PK2-GFP was not detectable [..] in day 14 oocysts prior to sporulation (Fig. 1B)", and "PK2-GFP was detectable in both midgut and salivary gland sporozoites (Fig. 1B)". However, no ookinetes of stage I-IV are shown, the non-sporulating day 14 oocyst shows a pancellular GFP signal, and it is unclear if the single depicted sporozoite is from midguts or salivary glands. Please clarify. The missing microscopy data could for example be included as supplementary images (as could be additional example images of each stage). On a side note, how did the authors define the apical tip of the sporozoite?

All microscopy figures: Please use black and white for single-channel images. This increases the contrast and facilitates the detection of distinct punctae. Also please indicate whether the depicted images are Z-stacks or single slices.

All of the insets lack a scale bar. Particularly in Stage V ookinetes in Fig 1B, but also in the other images they also do not really enlarge the image and provide not really additional insight. If you provide an inset, please make sure to adhere to good practices: Indicate in the original image where you placed the inset, enlarge so that it provides a useful magnification and add a scale bar.

Fig 3D: How many oocysts were quantified in each line?

Fig 3F: Some information is missing to judge the data. How many ookinetes were imaged per experiment, and in how many independent biological experiments? Over which time period were the ookinetes imaged and with which frame speed? 2 min appears quite short for ookinete motility, imaging ookinetes for 10-15 min with a frame rate of 15 sec/frame is more common in the field. Arrows indicating the apical tip of the ookinetes would be helpful to judge the speed and directionality. I am not sure if the WT ookinete is the best example of a gliding ookinete.

Fig 3G does not show Mean +- SEM, I suppose, given that it is a violin plot? They usually show mean/media plus confidence interval?

Fig 4: Could the authors please specify if the micronemes were quantified across the entire z stack or just in a single z slice for the expansion microscopy? Would that be a reason why the microneme numbers differ between expansion microscopy and EM? Are the numbers of micronemes comparable with those observed by others?

Suppl. Fig 1A/B: It would be good to indicate where the primers for the control PCR bind

Figure 1, Legend: "White boxes indicate zoom regions within image panels." is written twice.

Some abbreviations are not spelled out, e.g. PTD or CBP-O.

As a general statement, it would be helpful to add line numbers or at least page numbers to the manuscript to facilitate reviewing and referring to specific statements.

**2. Significance:**

PK2 is an important kinase essential for Plasmodium parasites during asexual development, while its function during mosquito stage development has not been investigated yet. The study attempts to do just that, with extensive imaging of PK2 localisation across the life cycle and solid phenotyping of a conditional knockout line, showing for the first time that PK2 is also important for mosquito infection. However, at the current state some conclusions, particularly regarding the presumed localisation of PK2 at the apical tip, are not well supported by the data. Indeed, for a function in microneme biogenesis and transport PK2 also doesn't necessarily need to localise there. The phenotypic analysis is also appears modest. A more precise pinpointing of the developmental arrest would help to understand PK2 function and would probably also be helpful to interpret the phosphoproteomics data.

Having addressed the above-mentioned points, this study will be interesting for basic parasitology research.

*Personal expertise:* Plasmodium molecular and cellular biology, focussing on mosquito-stage development.

**3. How much time do you estimate the authors will need to complete the suggested revisions:**

**Estimated time to Complete Revisions (Required)**

**(Decision Recommendation)**

Between 3 and 6 months

**4. *Review Commons* values the work of reviewers and encourages them to get credit for their work. Select 'Yes' below to register your reviewing activity at [Web of Science Reviewer Recognition Service](#) (formerly Publons); note that the content of your review will not be visible on Web of Science.**

**Web of Science Reviewer Recognition**

Yes

**Review #2**

**1.Evidence, reproducibility and clarity (Required)**

This study focuses on PK2, a Plasmodium kinase previously shown to be important for microneme secretion and host cell invasion in the P. falciparum blood stage. Here, Pashley et al evaluate the localization and function of PK2 in the parasite mosquito stages using the P. berghei rodent malaria model. Endogenous GFP tagging reveals that in addition to asexual blood stages, PK2 is also expressed in ookinetes, late oocysts and sporozoites but not gametocytes or zygotes, suggesting potential roles across Plasmodium invasive (zoite) stages. The authors evaluate PK2 localization using live, 3D-SIM and U-ExM fluorescence microscopy. While PK2 is generally distributed throughout the cell, an apical focus of signal is sometimes observed, although when present this appears to account for only a small fraction of the total signal and its relevance to the phenotypes observed is unclear. To investigate PK2 function during mosquito development, the authors replace the endogenous pk2 promoter with the ama1 promoter to generate a stage-specific knockdown. Interestingly, while ookinete conversion is not affected, oocyst and sporozoite formation is severely impacted. PK2 knockdown ookinetes have reduced motility and a decrease in apical micronemes, consistent with a possible defect in midgut invasion. Proteomic analysis shows a decrease in total levels of a few microneme proteins and altered phosphorylation levels in a range of proteins, including several in the IMC. Collectively, this work nicely demonstrates a critical role for PK2 in Plasmodium transmission, expanding its function beyond the blood stage; however, I have a few concerns about some of the conclusions. My specific comments are below:

**Major comments**

- The emphasis on PK2 concentration at the apical tip of ookinetes and sporozoites is not very compelling. While some images do appear to show a small apical focus of signal, it is not consistently observed (only 65% of ookinetes, no percent is given for sporozoite) and when present appears to be a very small fraction of the total signal. GFP signal appears generally dispersed throughout the cell with substantially higher concentrations often seen in the cell periphery or body (nucleus and elsewhere). For instance, the 3D-SIM "confirmation" of the apical focus is S1E shows greater levels of signal concentrated at the cell periphery about midway down the body of the ookinete. Perhaps the authors focus on this faint apical localization because of the microneme phenotype but given it is not consistently observed and represents such a small fraction of the total protein when it is, its relevance is unclear and the concluding statement of this section that "PK2-GFP is predominantly located at the apical end of all invasive stages" is simply not supported by the data shown. If the authors want to make these conclusions about apical concentration of PK2, stronger support is needed with some type of quantitative measure, especially given that the apical concentration when present appears to be very close to the signal level throughout the cell (as in the ookinete in 1B/C or S1D) or not obviously concentrated at all (as in the U-ExM images in 2C-F, where if anything, the most predominant puncta and highest concentration of puncta appears in the basal region).
- In the sporozoite imaging in figure 1, the authors have highlighted opposite ends of the same cell as "apical" in Fig 1B and C. In 1B, the boxed region appears to be the basal end with the signal correspond to some debris that is separate from the cell body in the DIC.
- Fig S1C: The authors observed a 58 kDa band which they conclude to be the predicted size of the tagged protein. However, a quick look at the protein sequence indicates 58 kDa is the predicted size of the protein without the GFP tag, which would add ~25 kDa. Shouldn't the PK2-GFP fusion be migrating at ~83 kDa?
- Fig 3I: the authors inject ookinetes to bypass midgut invasion and observe no salivary gland sporozoites resulting from pk2ptd ookinetes, concluding that PK2 has an additional role in sporozoite development. However, as only salivary gland sporozoites were examined, it is possible that hemocoel sporozoites are formed but fail to invade the salivary glands. The authors should either temper their conclusions or evaluate whether hemocoel sporozoites are formed.

**Minor comments**

- Fig S1A: the schematic makes it appear as if primer #1 is within the homology region in the plasmid.
- Fig 1B: the panel and legend should indicate that magenta is P28 for clarity. Also, what is the zoom magnification on the boxed regions?
- The manuscript indicate PK2-GFP expression was visible beginning in stage IV ookinetes but only stage V and VI

(mature) ookinetes are shown in 1B. To support this claim, the authors should include data demonstrating expression in stage IV.
- Fig S1B and S2B: it is not clear what the control PCR is in these gels. I assumed this is an amplicon from a different locus to ensure the quality of the WT DNA where no integration band is present. However, this appears not to be the case since the size of the control PCR amplicon in S2B is different between the WT and modified parasites.
- Fig 1B: the authors indicate that PK2-GFP expression was detectible in both midgut and salivary gland sporozoites but only a single sporozoite is. Which type of sporozoite this is?
- Fig S1F: to orient the reader, the entire cell should be shown, not just the boxed apical region. As presented, it is difficult to determine what the image is showing.
- Fig 2B: this U-ExM image of a blood stage schizont shows many foci of signal throughout the cell. An equivalent of two main foci near each nucleus is not obvious to me.
- The oocyst quantification data in Fig 3C and G would benefit from pie charts showing the prevalence of infection.
- The supplemental videos 1 and 2 mentioned in the text were not included with the manuscript, so I was not able to evaluate the motility data summarized in Fig 3F.
- Fig 5A: while qRT-PCR showed no difference in SOAP and CHT1 expression in pk2ptd parasites, protein levels were reduced. It might be interesting to perform IFAs to evaluate any changes in SOAP and CHT1 expression/localization (OPTIONAL).
- the reference to Patzewitz et al is sometimes given by the number 18 and sometimes by the author name/year.
- Punctuation: Remove apostrophe after "altered microneme positioning" in abstract. Period instead of comma at the end sentence "...pk2ptd parasites failed to infect susceptible mice".

**2. Significance:**

*Strengths:* The highlight of the paper is the clear demonstration of a critical, novel role for PK2 in the ookinete to oocyst transition. The impact on ookinete micronemes and motility suggests a midgut invasion defect. Taken with the previously reported blood stage merozoite phenotype and the stage-specific expression profile reported here, the results suggest PK2 is specifically important to the biology of Plasmodium invasive stages.

*Limitations:* The focus on the apical concentration of PK2 signal is tenuous and its relevance to the phenotype is unclear. The importance of PK2 in sporozoite biology remains unclear since ookinete injection experiments to bypass midgut invasion do not differentiate between defects in sporozoite development vs salivary gland invasion.

*Advance:* Plasmodium kinases have emerged as potential drug targets, yet the biology of these enzymes remains poorly understood, particularly outside of the vertebrate host. This study nicely demonstrates a critical role for PK2 in Plasmodium transmission, expanding its function beyond the blood stage.

*Audience:* this study will be of interest to the malaria field and to others broadly interested in kinase function and signaling in pathogenic protozoa.

*Reviewer expertise:* malaria genetics, biochemistry and cell biology

**3. How much time do you estimate the authors will need to complete the suggested revisions:**

**Estimated time to Complete Revisions (Required)**

**(Decision Recommendation)**

Between 1 and 3 months

**4. *Review Commons* values the work of reviewers and encourages them to get credit for their work. Select 'Yes' below to register your reviewing activity at Web of Science Reviewer Recognition Service (formerly Publons); note that the content of your review will not be visible on Web of Science.**

**Web of Science Reviewer Recognition**

No

**Reviewer #1**

**Evidence, reproducibility and clarity**

Summary
The authors here characterize the function of the protein kinase PK2 in Plasmodium berghei mosquito stages. They find that besides in asexual blood stages, PK2 is expressed in mature ookinetes, oocysts and sporozoites, with a putative localisation at the apical tip of the invasive stages. Conditional depletion of PK2 in mosquito-infecting stages results in slower ookinetes and an almost complete abolishment of oocyst formation which cannot be rescued by injecting ookinetes into the haemocoel, thus bypassing the midgut invasion step. Expansion and electron microscopy reveals a reduced number of micronemes in ookinetes, possibly explaining the gliding and invasion effect. The authors perform phosphoproteomics to determine targets of PK2 in ookinetes. PK2 is a major kinase of Plasmodium parasites and the characterisation of its role in mosquito stages is interesting. Yet the localisation of PK2 does currently not convincingly support an apical localisation. It is clear that PK2 is important for mosquito infection, however, its precise phenotype could be defined a bit more, and the phosphoproteomics are inconclusive with regards to the mechanism of action. Also, the data presentation should be improved to support their statements and to be transparent about the number of replicates in each experiment. Specific major and minor points of critique are indicated below.

Major points

1) Putative apical localisation of PK2

At the end of the second result section, the authors summarize their findings with "Collectively, these GFP fluorescence data indicate that PK2-GFP is predominantly located at the apical end of all invasive stages". Yet, this statement is not supported by the presented data: In all presented stages, the majority of the GFP signal is found in the cytoplasm or partially in the nucleus, with different levels of speckles. There is no apical localisation visible in schizonts. While there might be an apical punctuate signal in some ookinetes, the images and their quantification are not yet convincing, and "predominantly apical localisation" does not hold true. This is especially true for the expansion microscopy images, where the GFP signal looks very noisy. The authors should present more images than the single example image they currently show for each stage and microscopy technique to support their statements about localisation. They should also state clear criteria how they define and quantify "apical localisation" and provide absolute numbers how many images were taken and analysed per stage in addition to percentages. One way

to quantify would be to draw lines across either the apical tip or randomly selected lateral points of their target cells and plot GFP intensity profiles (e.g. in relation to MyoA-mCherry). Imaging with less background (e.g. by generating a new cell line, see the next point) and providing images of wildtype parasites stained with the same conditions (especially for Expansion Microscopy) to control for unspecific antibody binding and noise could also help to provide more convincing localisation data.

*We thank the reviewer for these suggestions. To address this issue, we have undertaken additional live cell imaging (Figure 1) and provide a new Figure 2 where we used Structured Illumination Microscopy (SIM) to clarify the location of the protein. In this figure, we show clearly that PbPK2-GFP is located at distinct puncta throughout the parasite body, but it is enriched at both the apical and basal end of the cell. The expansion data have been moved to Supplementary Figure 2, and we state that U-ExM provided no distinct apical localization (line 175-177).*

Importantly, the Western blot presented in Suppl Fig 1C suggests a considerable amount of cleaved GFP, possibly confounding the localisation and partially explaining the pan-cellular distribution of the signal. The authors should comment in their results that the localisation could be partially confounded by cleaved GFP. A better, albeit more work intensive solution would be to generate a new parasite line testing either a different linker between the GFP and PK2 or using an epitope tag, e.g. FLAG or HA, combined with immunofluorescence assays, to test the localisation of PK2. FLAG/HA-Tags might also help to reduce the speckled background in expansion microscopy.

*We agree with the reviewer on this point, and we have provided a new Western blot image (Supplementary Figure 1C). Although it is likely that creation of parasite lines expressing a FLAG- or HA-tagged PK2 and their use in an immunofluorescence assay would reduce background, we believe that this work is outside the scope of this study. It would take a considerable amount of time to complete, involve the use of additional mice, and require a person to do the work. This is not feasible because the PDRA who performed the experiments has finished their contract, and there is no certainty that this alternative approach would provide better data.*

If new experiments and data analysis do not show convincingly a consistent PK2-GFP localisation at the apical tip of all stages, the authors should tone down their conclusion. PK2 may well have a function in microneme formation/secretion without being localised at the apical tip.

*We agree with the reviewer that the location of PK2 was unclear. Now we have performed SIM imaging and this technique reveals a clear apical location, as shown in new Figure 2. We provide quantitative data with the same parasite line using live cell imaging (lines 131-133 in the manuscript). We also provide more convincing live cell imaging figures showing apical localization (Figure 1), and representative examples of PbPK2-GFP location in Supplementary Figure 1.*

Fig 2B: The authors write "The PK2-GFP signal showed limited overlap with that of the intranuclear spindle microtubules, indicating that PK2 is extra-nuclear and not a major component of the mitotic spindle apparatus in schizonts (Fig. 2B)." How are the authors sure that these are intranuclear spindles? To me these tubulin structures do not look like spindles, but like the subpellicular microtubules surrounding the forming merozoites (in line with the non-nuclear localisation of these tubulin structures e.g. in the lower right merozoite).

*We thank the reviewer for this important suggestion. To avoid any confusion, we have removed this interpretation from the manuscript and the associated figure is now part of Supplementary Figure 2.*

2) PK2 knockdown phenotype

While ookinetes seem to be a bit slower, this phenotype is not sufficient to explain the total absence of oocysts, as also other slow-moving ookinetes successfully invade mosquito midguts, at least to some extend (see e.g. PMID: 27084458, 35506479). Thus, PK2 likely has another phenotype either for ookinete-to-oocyst transition or for oocyst growth. Indeed, when the authors bypass the midgut traversal step, PK2-knockdown parasites still do not form sporozoites, indicating that midgut traversal is not the (only) limiting factor of the PK2 knockdown. Do the parasites form oocysts after hemocoel injection, and do these grow and sporulate? This would be important to conclude if PK2 is already essential for ookinete-to-oocyst conversion, if it is important for oocyst growth or instead only for sporozoite formation. Right now the data seems to points towards ookinete-to-oocyst transition, which would not be invasion and thus not be related to secretory vesicles.

*We agree with the reviewer. The data suggest that the major defects are in ookinete motility and ookinete to oocyst transition, since few oocysts were observed. We have repeated the ookinete motility assay using a 10 mins window (instead of the 2 mins used previously). The data show a clear inability of ookinetes to move (Figure 3F). We also confirmed its function in sporozoite formation by using direct ookinete injection into the haemocoel to bypass*

*midgut traversal (Figure 3I). No sporogony and no sporozoite formation were observed. Directly injected ookinetes developing into sporozoites would have been detected and counted (line 218-223).*

Once they have pinpointed the point of arrest of the knockout better, the authors could further streamline the discussion and using more precise language depending on the actual phenotype (for example, the authors currently state that PK2 is essential for oocyst viability, and they also state that PK2 has a role in sporozoite development, when so far the data only shows that PK2 is essential for oocyst formation with some evidence that it is important for ookinete motility and microneme formation).

*As requested by the reviewer, we have streamlined the manuscript and placed more emphasis on ookinete-oocyst transition throughout the relevant sections in the Results (line 222), and Discussion. We have removed any reference to oocyst viability.*

The proteome and phosphoproteome are unfortunately not very informative regarding the mechanism of action of PK2. Do the authors have an explanation why the (I assume) asexual protein EMAP1 is such a significant hit for hypophosphorylation? Is this a target of PK2 in blood stages? Generally, how much does the phosphoproteome of PK2-PTD in ookinetes overlap with the published blood-stage phosphoproteome of PK2 depletion? One would assume that at least some of the targets are conserved. How did the authors purify ookinetes and could it be that residual asexual parasites in the culture (which still express PK2) confound the data? Could that explain why PK2 does not appear in the proteome as down-regulated? Without robust downregulation of the targeted protein, it is difficult to draw conclusions about other deregulated proteins. The authors should also state somewhere how many replicates were done for the proteome and the phosphoproteome.

*We thank the reviewer for this important suggestion. We have toned down the interpretation of the phosphoproteome data, stating that it gave "some mechanistic insight into how PK2 may have key roles in transmission-stage development." (line 325). We have removed reference to EMAP1 and focused on ookinete-associated proteins. Additionally, we compare our data to the phosphoproteome data of schizont stages (Rawat et al. 2023), and only two proteins (PBANKA_1342300 - conserved gene of unknown function, and PBANKA_1423500 - 60S ribosomal protein L13-2, putative) are present in the two datasets. This suggests alternative roles for phosphoregulation in these two stages, as discussed (line 339). We state that experiments were performed using three biological replicates of each parasite line (line 566 in Methods and in the legend of Figure 5 – lines 767 and 770).*

3) Data presentation and statistics

Using a bar graph and SEM is not appropriate for data with only two data points/biological replicates (e.g. Fig 3E, I, Suppl Fig. 2C-F). Showing individual values (as scatter plot) would be more appropriate. Also, how did the authors do statistics on only two replicates in Suppl fig. 2C, D? Were indeed technical and biological replicates pooled to obtain n=4 as the text in the statistical analysis section seems to suggest? This would not be correct as the error between technical replicates is much smaller that the variation between biological replicates. To perform statistical analysis, at least 3, better more, biological replicates (each the mean of appropriate number of technical replicates) are required. Alternatively, the authors can just depict the individual results of the biological replicates as a scatter plot and leave it to the reader to judge if this is a clear deregulation of expression. Please also indicate to what the expression is normalised. I would recommend scatter plots also for all experiments with only 3 data points (e.g. 3B, H). For Fig 3E, does independent experiments mean independent mosquito cages or just two mosquitoes?

*We thank the reviewer for raising these important points. We agree that individual biological replicate values should be shown, particularly for experiments with small sample sizes. We have therefore revised Fig. 3B, E, H and I to display the individual average biological replicate values on the bar graphs and revised Supplementary Figs. 3C-F to show only averages of the technical replicates.*

*For Fig. 3E, the experiment was performed on three independent occasions. Each replicate consisted of a separate bite-back experiment using 20 mosquitoes. This is now clarified in the figure legend: the three data points are for three independent transmission experiments, rather than for individual mosquitoes.*

*For Fig. 3I, haemocoel injection was performed using either 800 or 1,600 ookinetes, with each condition examined in two independent experiments.*

*Supplementary Fig. 3C-F originally showed qRT–PCR data generated from two independent biological replicates, each measured using two technical replicates. We have since repeated the experiment and have combined the data in updated Supplementary Figs. 3C-F to reflect this. We have also updated the methods section accordingly. The figure legends also now state that qRT–PCR expression data were normalised to control genes arginyl-tRNA synthetase (PBANKA_1434200) and hsp70 (PBANKA_0818900).*

Minor points

Ookinete stages: There is no consensus in the field about how many distinct "stages" an ookinete undergoes during maturation (it is likely a continuous process, anyways). While

Siciliano et al (PMID 32256458) define five stages and Darif et al (PMID 41025598) define seven stages, the authors divide maturation here into six stages. Which criteria did the authors use to define the stages in their images? If the ookinete staging here really is important for localisation and characterisation of PK2 (one could argue it is not), the authors should include a scheme and example images in the supplements, otherwise just forgo any staging and use the wording early and late retorts with respective example images.

*We thank the reviewer for this suggestion. We used criteria set out by Janse et al (ref 9 – and many of our other publications) and we have updated the methods section to reflect this (line 431). We have also included images depicting PbPK2-GFP expression through zygote-ookinete transition in Figure 1.*

Fig 1B: The authors refer to Fig 1B for various statements regarding PK2-GFP expression and localisation, e.g.: "No detectable GFP signal was observed [...] in zygotes during early ookinete differentiation (stages I-III) (Fig. 1B)", "PK2-GFP was visible in stage IV ookinetes ", "PK2-GFP was not detectable [..] in day 14 oocysts prior to sporulation (Fig. 1B)", and "PK2-GFP was detectable in both midgut and salivary gland sporozoites (Fig. 1B)". However, no ookinetes of stage I-IV are shown, the non-sporulating day 14 oocyst shows a pancellular GFP signal, and it is unclear if the single depicted sporozoite is from midguts or salivary glands. Please clarify. The missing microscopy data could for example be included as supplementary images (as could be additional example images of each stage). On a side note, how did the authors define the apical tip of the sporozoite?

*We thank the reviewer for this suggestion. We have now included images depicting PbPK2-GFP expression through the zygote-ookinete transition in Figure 1. We have also clarified descriptions of GFP fluorescence in the results section, as well as stating in the text that sporozoites were taken from the salivary glands (line 140). In addition, the apical tip of the sporozoite was defined morphologically as the tapered end of the crescent-shaped cell.*

All microscopy figures: Please use black and white for single-channel images. This increases the contrast and facilitates the detection of distinct punctae. Also please indicate whether the depicted images are Z-stacks or single slices.

*While we appreciate the reviewer's request to use black and white for all microscopy images, we feel that this is unnecessary and our revised Figure 1 and 2 clearly show localization of GFP fluorescence. Our images in Figure 4 clearly show tubulin staining and microneme-like structures using NHS-Ester. Figures 1 and 2 are single slice images, not Z-stacks. Figure 4 shows representative images, taken from maximum-intensity projections.*

*All U-ExM images have now been moved to Supplementary Figure 2 since they show no clear GFP enrichment.*

All of the insets lack a scale bar. Particularly in Stage V ookinetes in Fig 1B, but also in the other images they also do not really enlarge the image and provide not really additional insight. If you provide an inset, please make sure to adhere to good practices: Indicate in the original image where you placed the inset, enlarge so that it provides a useful magnification and add a scale bar.

*We thank the reviewer for this suggestion. We have now included scale bars on all microscopy images, and clearly highlighted with white boxes which parts of the ookinetes have been used for the insets.*

Fig 3D: How many oocysts were quantified in each line?

*For WT-GFP lines, 10 oocysts were analysed in 3 independent experiments (total n = 30). For pk2ptd parasites, a total of 11 oocysts were detected across the three experiments, all of which were analysed.*

Fig 3F: Some information is missing to judge the data. How many ookinetes were imaged per experiment, and in how many independent biological experiments? Over which time period were the ookinetes imaged and with which frame speed? 2 min appears quite short for ookinete motility, imaging ookinetes for 10-15 min with a frame rate of 15 sec/frame is more common in the field. Arrows indicating the apical tip of the ookinetes would be helpful to judge the speed and directionality. I am not sure if the WT ookinete is the best example of a gliding ookinete.

*We have now performed the ookinete motility assay for 10 min (Figure 3F, Supplementary videos 1 and 2) as accepted in the field.*

Fig 3G does not show Mean +- SEM, I suppose, given that it is a violin plot? They usually show mean/media plus confidence interval?

*We thank the reviewer for highlighting this. We have changed the description of the statistic accordingly in the figure legends.*

Fig 4: Could the authors please specify if the micronemes were quantified across the entire z stack or just in a single z slice for the expansion microscopy? Would that be a reason why the microneme numbers differ between expansion microscopy and EM? Are the numbers of micronemes comparable with those observed by others?

*For U-ExM, the micronemes were quantified across maximum-intensity projections, and we have updated the methods section to reflect this (line 510-516). The TEM does not have Z slices and hence the number of micronemes can differ compared to the U-ExM results; however, the overall observation for both approaches was a decrease in microneme numbers at the apical end.*

Suppl. Fig 1A/B: It would be good to indicate where the primers for the control PCR bind

*We have included this.*

Figure 1, Legend: "White boxes indicate zoom regions within image panels." is written twice.

*This has been amended.*

Some abbreviations are not spelled out, e.g. PTD or CBP-

*We thank the reviewer for this suggestion and have revised the manuscript accordingly.*

As a general statement, it would be helpful to add line numbers or at least page numbers to the manuscript to facilitate reviewing and referring to specific statements.

*We have added the number lines*

**Significance**

PK2 is an important kinase essential for Plasmodium parasites during asexual development, while its function during mosquito stage development has not been investigated yet. The study attempts to do just that, with extensive imaging of PK2 localisation across the life cycle and solid phenotyping of a conditional knockout line, showing for the first time that PK2 is also important for mosquito infection. However, at the current state some conclusions, particularly regarding the presumed localisation of PK2 at the apical tip, are not well supported by the data. Indeed, for a function in microneme biogenesis and transport PK2 also doesn't necessarily need to localise there. The phenotypic analysis is also appears modest. A more precise pinpointing of the developmental arrest would help to understand PK2 function and would probably also be helpful to interpret the phosphoproteomics data.
Having addressed the above-mentioned points, this study will be interesting for basic parasitology research.

*We provide new revised Figures 1 and 2 to give a clearer apical localization using SIM microscopy. The motility assay has been repeated and performed over a 10 min period. An additional revision on the developmental arrest is explained in the text. We hope that these changes clarify the work and will be helpful to the reviewers based on their queries.*

**Reviewer #2 (Evidence, reproducibility and clarity (Required)):**

This study focuses on PK2, a Plasmodium kinase previously shown to be important for microneme secretion and host cell invasion in the P. falciparum blood stage. Here, Pashley et al evaluate the localization and function of PK2 in the parasite mosquito stages using the P. berghei rodent malaria model. Endogenous GFP tagging reveals that in addition to asexual blood stages, PK2 is also expressed in ookinetes, late oocysts and sporozoites but not gametocytes or zygotes, suggesting potential roles across Plasmodium invasive (zoite) stages. The authors evaluate PK2 localization using live, 3D-SIM and U-ExM fluorescence microscopy. While PK2 is generally distributed throughout the cell, an apical focus of signal is sometimes observed, although when present this appears to account for only a small fraction of the total signal and its relevance to the phenotypes observed is unclear. To investigate PK2 function during mosquito development, the authors replace the endogenous pk2 promoter with the ama1 promoter to generate a stage-specific knockdown. Interestingly, while ookinete conversion is not affected, oocyst and sporozoite formation is severely impacted. PK2 knockdown ookinetes have reduced motility and a decrease in apical micronemes, consistent with a possible defect in midgut invasion. Proteomic analysis shows a decrease in total levels of a few microneme proteins and altered phosphorylation levels in a range of proteins, including several in the IMC. Collectively, this work nicely demonstrates a critical role for PK2 in Plasmodium transmission, expanding its function beyond the blood stage; however, I have a few concerns about some of the conclusions. My specific comments are below:

Major comments

-The emphasis on PK2 concentration at the apical tip of ookinetes and sporozoites is not very compelling. While some images do appear to show a small apical focus of signal, it is not consistently observed (only 65% of ookinetes, no percent is given for sporozoite) and when present appears to be a very small fraction of the total signal. GFP signal appears generally dispersed throughout the cell with substantially higher concentrations often seen in the cell periphery or body (nucleus and elsewhere). For instance, the 3D-SIM "confirmation" of the apical focus is S1E shows greater levels of signal concentrated at the cell periphery about midway down the body of the ookinete. Perhaps the authors focus on

this faint apical localization because of the microneme phenotype but given it is not consistently observed and represents such a small fraction of the total protein when it is, its relevance is unclear and the concluding statement of this section that "PK2-GFP is predominantly located at the apical end of all invasive stages" is simply not supported by the data shown. If the authors want to make these conclusions about apical concentration of PK2, stronger support is needed with some type of quantitative measure, especially given that the apical concentration when present appears to be very close to the signal level throughout the cell (as in the ookinete in 1B/C or S1D) or not obviously concentrated at all (as in the U-ExM images in 2C-F, where if anything, the most predominant puncta and highest concentration of puncta appears in the basal region).

*We thank the reviewer for these suggestions. To address this issue, we have undertaken additional live cell imaging (Figure 1) and provide a new Figure 2 where we used Structured Illumination Microscopy (SIM) to clarify the location of the protein. In this figure, we show clearly that PbPK2-GFP is located at distinct puncta throughout the parasite body, but it is enriched at both the apical and basal end of the cell. The expansion data have been moved to Supplementary Figure 2, and we state that U-ExM provided no distinct apical localization (line 175-177).*

-In the sporozoite imaging in figure 1, the authors have highlighted opposite ends of the same cell as "apical" in Fig 1B and C. In 1B, the boxed region appears to be the basal end with the signal correspond to some debris that is separate from the cell body in the DIC.

*We thank the reviewer and have changed the sporozoite figure to better represent the apical end of the sporozoite (Figure 1).*

-Fig S1C: The authors observed a 58 kDa band which they conclude to be the predicted size of the tagged protein. However, a quick look at the protein sequence indicates 58 kDa is the predicted size of the protein without the GFP tag, which would add ~25 kDa. Shouldn't the PK2-GFP fusion be migrating at ~83 kDa?

*We agree with the reviewer on this point, and we have provided a new Western blot image (Supplementary Figure 1C).*

-Fig 3I: the authors inject ookinetes to bypass midgut invasion and observe no salivary gland sporozoites resulting from pk2ptd ookinetes, concluding that PK2 has an additional role in sporozoite development. However, as only salivary gland sporozoites were examined, it is possible that hemocoel sporozoites are formed but fail to invade the salivary

glands. The authors should either temper their conclusions or evaluate whether hemocoel sporozoites are formed.

*We thank the reviewer for this important observation. We agree that examination of salivary-gland sporozoites alone does not distinguish between a failure to produce sporozoites and a defect in the migration of haemocoel sporozoites to, or invasion of, the salivary glands. We have therefore revised the manuscript to avoid concluding that PK2 has a direct role in sporozoite development.*

*The haemocoel-injection experiment demonstrates that bypassing traversal of the mosquito midgut is insufficient to restore the production of salivary-gland sporozoites by pk2ptd parasites. This finding supports an additional requirement for PK2 downstream of midgut traversal, but the precise affected process cannot be determined from the data we have presented.*

*We have clarified this limitation in the Results and Discussion and have revised the Abstract accordingly. We now state that PK2 is required for parasite development downstream of midgut traversal, rather than specifically for sporozoite development.*

Minor comments

-Fig S1A: the schematic makes it appear as if primer #1 is within the homology region in the plasmid.

*We have revised the figure to clearly show that primer one binds within the pk2 gene.*

-Fig 1B: the panel and legend should indicate that magenta is P28 for clarity. Also, what is the zoom magnification on the boxed regions?

*We thank the reviewer for this suggestion. We have made substantial changes to Figure 1 but have added in the legend that P28 is magenta.*

-The manuscript indicate PK2-GFP expression was visible beginning in stage IV ookinetes but only stage V and VI (mature) ookinetes are shown in 1B. To support this claim, the authors should include data demonstrating expression in stage IV.

*We thank the reviewer for this suggestion and have updated Figure 1 to show all stages of ookinete development, clearly showing that stage 5 and stage 6 are the only stages that express PK2-GFP.*

-Fig S1B and S2B: it is not clear what the control PCR is in these gels. I assumed this is an amplicon from a different locus to ensure the quality of the WT DNA where no integration

band is present. However, this appears not to be the case since the size of the control PCR amplicon in S2B is different between the WT and modified parasites.

*We have updated the Supplementary figure to highlight which primer combination represents which parasite line.*

-Fig 1B: the authors indicate that PK2-GFP expression was detectible in both midgut and salivary gland sporozoites but only a single sporozoite is. Which type of sporozoite this is?

*We thank the reviewer for highlighting this. The sporozoite illustrated in the figure is from salivary glands.*

-Fig S1F: to orient the reader, the entire cell should be shown, not just the boxed apical region. As presented, it is difficult to determine what the image is showing.

*We have substantially revised this figure and a new Supplementary figure 1 is presented.*

-Fig 2B: this U-ExM image of a blood stage schizont shows many foci of signal throughout the cell. An equivalent of two main foci near each nucleus is not obvious to me.

*We have moved this figure to the supplementary section and clarified in the text that the U-ExM localization of PK2-GFP expression is inconclusive. Instead, we replaced this with a new Figure 2 using SIM microscopy showing a much clearer apical and basal localization.*

-The supplemental videos 1 and 2 mentioned in the text were not included with the manuscript, so I was not able to evaluate the motility data summarized in Fig 3F.

*We apologize to the reviewer for this oversight. We have now included videos for both.*

Reviewer #2 (Significance (Required)):

Strengths: The highlight of the paper is the clear demonstration of a critical, novel role for PK2 in the ookinete to oocyst transition. The impact on ookinete micronemes and motility suggests a midgut invasion defect. Taken with the previously reported blood stage merozoite phenotype and the stage-specific expression profile reported here, the results suggest PK2 is specifically important to the biology of Plasmodium invasive stages.

Limitations: The focus on the apical concentration of PK2 signal is tenuous and its relevance to the phenotype is unclear. The importance of PK2 in sporozoite biology remains unclear since ookinete injection experiments to bypass midgut invasion do not

differentiate between defects in sporozoite development vs salivary gland invasion.

Advance: Plasmodium kinases have emerged as potential drug targets, yet the biology of these enzymes remains poorly understood, particularly outside of the vertebrate host. This study nicely demonstrates a critical role for PK2 in Plasmodium transmission, expanding its function beyond the blood stage.

September 11, 2026

RE: Life Science Alliance Manuscript #LSA-2026-03863

Prof. Rita Tewari
University of Nottingham
School of Life Sciences
Queens Medical Centre
Nottingham NG7 2UH
United Kingdom

Dear Dr. Tewari,

Thank you for submitting your revised manuscript entitled "Plasmodium Protein Kinase 2 is required for ookinete to oocyst transition, and parasite transmission by the mosquito.". We sought comments from both of the original reviewers from Review Commons, however only Reviewer 2 was available to evaluate your revised manuscript. Thank you for your patience during the re-review process. We appreciate the detailed comments from this reviewer who is overall supportive of publication. We concur that the text description of PK2 localization should be adjusted to more closely reflect the images shown in Figures 1 and S1. We would be happy to publish your paper in Life Science Alliance pending these text adjustments and final revisions necessary to meet our formatting guidelines.

MANUSCRIPT ORGANIZATION AND FORMATTING:

To avoid unnecessary delays in the acceptance and publication of your paper, please read the following information carefully. Full guidelines are available on our Instructions for Authors page, https://www.life-science-alliance.org/authors

**Submission of a paper that does not conform to Life Science Alliance guidelines will delay the acceptance of your manuscript.**

-Please upload your main manuscript text as an editable doc file.
-Please upload your main and supplementary figures as single files.
-Please add a Running Title in our system.
-Please add a Summary Blurb/Alternate Abstract in our system.
-Please add a Category for your manuscript in our system.
-Please add the X and Bluesky handles of your host institute/organization, as well as your own, and/or one of the authors, in our system.
-The titles in both the system and the manuscript file must be consistent with each other.
-Please be sure that the author order is correct and matches between the system and manuscript file.
-Please add Author Contributions for each author in our system as well.
-Please move your main, supplementary figure, video, and table legends in the main manuscript text after the references section.
-Please add a Conflict of Interest statement to your main manuscript text. If there is no COI, please use the following formulation: "The authors declare that they have no conflict of interest."
-Please add callouts for Figure S2A-F to your main manuscript text.
-Please ensure full details are included in the Live Imaging section of your methods, including illumination source, stage temperature, and acquisition interval.

We encourage our authors to provide original source data, particularly uncropped/-processed electrophoretic blots and spreadsheets for the main figures of the manuscript. If you would like to add source data, we would welcome one PDF/Excel-file per figure for this information. These files will be linked as supplementary "Source Data" files.

It is Life Science Alliance policy that if requested, original data images must be made available to the editors. Failure to provide original images upon request will result in unavoidable delays in publication. Please ensure that you have access to all original data images prior to final submission.

If you are planning a press release on your work, please inform us immediately to allow informing our production team and scheduling a release date.

We welcome submissions of potential cover images for the issue of LSA in which your work would appear. If you have high quality images associated with this work, please feel free to email these, with a caption, to the journal office.

LSA encourages authors to provide a 30-60 second video where the study is briefly explained. These videos will be appear embedded with the manuscript online at Life Science Alliance, and on social media to promote the published paper and authors (for examples, see https://docs.google.com/document/d/1-UWCfbE4pGcDdcgzcmiuJl2XMBJnxKYeqRvLLrLSo8s/edit?usp=sharing). Corresponding or first-authors are welcome to submit the video. Please submit only one video per manuscript. The video can be emailed to contact@life-science-alliance.org

FINAL FILES:

To upload the final version of your manuscript, please log in to your account: https://lsa.msubmit.net/cgi-bin/main.plex
You will be guided to complete the submission of your revised manuscript and to fill in all necessary information. Please get in touch in case you do not know or remember your login name.

The following items are required for acceptance.

-- An editable version of the final text (.DOC or .DOCX) is needed for copyediting (no PDFs).

-- High-resolution figure, supplementary figure and video files uploaded as individual files: See our detailed guidelines for preparing your production-ready images, https://www.life-science-alliance.org/authors

-- Summary blurb (enter in submission system): A short text summarizing in a single sentence the study (max. 200 characters including spaces). This text is used in conjunction with the titles of papers, hence should be informative and complementary to the title. It should describe the context and significance of the findings for a general readership; it should be written in the present tense and refer to the work in the third person. Author names should not be mentioned.

**The license to publish form must be signed before your manuscript can be sent to production. A link to the license to publish form will be available to the corresponding author only. Please take a moment to check your funder requirements.**

**Reviews, decision letters, and point-by-point responses associated with peer-review at Life Science Alliance will be published, alongside the manuscript. If you do want to opt out of having the reviewer reports and your point-by-point responses displayed, please let us know immediately.**

Thank you for your attention to these final processing requirements. Please revise and format the manuscript and upload materials as soon as you are able.

Thank you for this interesting contribution to the literature. We look forward to publishing your paper in Life Science Alliance.

Sincerely,

Tim Fessenden
Scientific Editor
Life Science Alliance

------------------------------------------------------------------------------
Reviewer #2 (Comments to the Authors (Required)):

The authors have thoughtfully responded to my comments and provided new data that address many of my concerns. The study provides clear evidence that PK2 is required for the ookinete to oocyst transition and likely performs an additional important function after midgut penetration. This novel PK2 function in Plasmodium transmission will be of significant interest. While the new imaging data and quantification help to clarify PK2 localization and substantially improve the manuscript, I still have a few concerns about the description and interpretation of the data. My specific comments on the revised manuscript are below:

-line 108: since some merozoites in Fig 1A appear to show >2 foci (also seen in Fig 2A), the title to this section should be adjusted to reflect the description in the text by removing the word "two". Otherwise, some type of quantification is needed.

-lines 126-134: this section makes no mention that the majority of the GFP signal is dispersed throughout the cell body. This need clarification since it otherwise reads as if signal is only or most prominently at the apical and/or basal ends, which is not the case. The transition to a "more distributed pattern along the parasite body" in Stage VI is not apparent to me: signal is similarly distributed in the stage V and VI images with the only obvious difference being the size of the structure assumed to be the contractile ring and perhaps a slight increase in signal at the apical end. The "discrete focus at the apical end" is very subtle in the Stage V image being referred to in Fig 1B,C. A much more prominent/discrete focus is seen in the in the center of the cell but is not mentioned. The images in Fig S1D all show GFP concentrated at the nucleus but this is also not mentioned. The apical focus is more compelling in the 3D SIM images, but there are many foci with similar or greater signal within the cell, often

around the nucleus, and these are never mentioned in the ookinete images (or sporozoites image in Fig 1, which shows a similar patten). The authors describe the internal foci that are often proximal to the nucleus in the blood stages - why are they not mentioned in the other stages? I assume the authors focus on the apical signal because of the microneme positioning and motility phenotypes, but it seems equally possible that PK2 controls these processes from other sites in the cell.

-line 144-145: the statement that "PK2-GFP is predominantly located at the apical end" is simply not supported by the data. Predominantly indicates the main or most noticeable feature.

-Fig 2B: The schematic of the parasite apex implies more about PK2 localization than the images actually demonstrate. If the authors want to imply localization to the apical polar rings or conoidal body, then co-markers are needed. Without this, it would be better to mention this as possibility in the text without a cartoon that places PK2 at this site.

-line 297: "particular concentration at the apical tip" is misleading. The apical signal is minor with most signal concentrated within the cell near the nucleus.

-Lines 312-318: these two sentences are with different interpretations of the strength of the conclusion. Line 314: PK2 also has an additional function... vs Line 318: may also have an additional later role.

-Fig S1A: the schematic still seems to indicate that primer #1 anneals within the homology region in the plasmid, not outside in the genome.

**Reviewer #1**

-line 108: since some merozoites in Fig 1A appear to show >2 foci (also seen in Fig 2A), the title to this section should be adjusted to reflect the description in the text by removing the word "two". Otherwise, some type of quantification is needed.

*We agree with the reviewer and have revised the title to "PbPK2 localises to discrete foci throughout asexual blood-stage development". (line 108).*

-lines 126-134: this section makes no mention that the majority of the GFP signal is dispersed throughout the cell body. This need clarification since it otherwise reads as if signal is only or most prominently at the apical and/or basal ends, which is not the case. The transition to a "more distributed pattern along the parasite body" in Stage VI is not apparent to me: signal is similarly distributed in the stage V and VI images with the only obvious difference being the size of the structure assumed to be the contractile ring and perhaps a slight increase in signal at the apical end. The "discrete focus at the apical end" is very subtle in the Stage V image being referred to in Fig 1B,C. A much more prominent/discrete focus is seen in the in the center of the cell but is not mentioned. The images in Fig S1D all show GFP concentrated at the nucleus but this is also not mentioned. The apical focus is more compelling in the 3D SIM images, but there are many foci with similar or greater signal within the cell, often around the nucleus, and these are never mentioned in the ookinete images (or sporozoites image in Fig 1, which shows a similar patten). The authors describe the internal foci that are often proximal to the nucleus in the blood stages - why are they not mentioned in the other stages? I assume the authors focus on the apical signal because of the microneme positioning and motility phenotypes, but it seems equally possible that PK2 controls these processes from other sites in the cell.

*We thank the reviewer for highlighting this. We have revised the text to say:*

*"At stage V, PbPK2-GFP became readily detectable throughout the developing ookinete, with a broadly distributed intracellular signal together with several regions of focal enrichment. These included prominent internal foci, frequently in proximity to the nucleus, as well as enrichment at the apical and basal ends in a subset of parasites (Fig. 1B,C; Supplementary Fig. 1D). A structure resembling the developing contractile ring was also apparent in some cells. In mature ookinetes (stage VI), PbPK2-GFP remained distributed throughout the parasite body with multiple discrete intracellular foci." (Lines 126-133).*

*We have also revised the text in the section describing our SIM microscopy to read "including multiple internal foci frequently concentrated around the nucleus, together with regions of enrichment near the cell periphery and at the poles." (lines 163-165).*

-line 144-145: the statement that "PK2-GFP is predominantly located at the apical end" is simply not supported by the data. Predominantly indicates the main or most noticeable feature.

*We agree with the reviewer and have revised the text to state:*

*"Collectively, these GFP fluorescence data indicate that PK2-GFP is expressed in the invasive mosquito stages and displays a broadly distributed, punctate localisation pattern with variable polar enrichment." (lines 145-148)*

-Fig 2B: The schematic of the parasite apex implies more about PK2 localization than the images actually demonstrate. If the authors want to imply localization to the apical polar rings or conoidal body, then co-markers are needed. Without this, it would be better to mention this as possibility in the text without a cartoon that places PK2 at this site.

*We thank the reviewer for making this point. We have removed the schematic to avoid any confusion around the overall localization of PK2 in mature ookinete stages.*

-line 297: "particular concentration at the apical tip" is misleading. The apical signal is minor with most signal concentrated within the cell near the nucleus.

*We agree with the reviewer and have revised the text to state:*

*"PK2 was also expressed in sporozoites, displaying a distributed intracellular pattern with prominent internal/perinuclear signal and variable enrichment toward the apical tip." (lines 299-300).*

-Lines 312-318: these two sentences are with different interpretations of the strength of the conclusion. Line 314: PK2 also has an additional function... vs Line 318: may also have an additional later role.

*We agree that the wording was inconsistent. We have revised these sentences to use consistent language and to distinguish between the evidence for a post-midgut requirement for PK2 and the uncertainty regarding its precise later function. The revised text now states:*

*"This further supports an additional requirement for PK2 after midgut traversal, although the precise developmental stage and function remain unclear" (lines 319-321).*

-Fig S3A: the schematic still seems to indicate that primer #1 anneals within the homology region in the plasmid, not outside in the genome.

*We thank the reviewer for this comment. Primer 1 anneals to the endogenous genomic locus outside the sequence represented in the C-terminal tagging construct. The schematic is intended to show the endogenous locus, the tagging construct, and the correctly recombined locus, and does not depict the full plasmid backbone or the precise boundaries of the cloned homology arm. We have revised the figure legend to make this distinction clearer and to indicate explicitly that primer 1 is genomic and lies outside the targeting construct, stating "Primer 1 is the integration*

*primer that anneals from the genomic sequence outside of the homology arm of the plasmid, thereby indicating correct integration." (Lines 784-786).*

September 21, 2026

RE: Life Science Alliance Manuscript #LSA-2026-03863R

Prof. Rita Tewari
University of Nottingham
School of Life Sciences
Queens Medical Centre
Nottingham NG7 2UH
United Kingdom

Dear Dr. Tewari,

Thank you for submitting your Research Article entitled "Plasmodium Protein Kinase 2 is required for ookinete to oocyst transition and parasite transmission". It is a pleasure to let you know that your manuscript is now accepted for publication in Life Science Alliance. Congratulations on this interesting work.

The final published version of your manuscript will be deposited by us to PubMed Central upon publication.

Your manuscript will now progress through copyediting and proofing. During the proofing stage, please provide additional details in the "Life Imaging" section of your Materials and Methods to indicate illumination source, microscope temperature, and imaging interval.

Your article will publish open access upon publication under a CC-BY license.

Reviews, decision letters, and point-by-point responses associated with peer-review at Life Science Alliance will be published, alongside the manuscript. If you do want to opt out of having the reviewer reports and your point-by-point responses displayed, please let us know immediately.

***IMPORTANT: If you will be unreachable at any time, please provide us with the email address of an alternate author. Failure to respond to routine queries may lead to unavoidable delays in publication.***

Scheduling details will be available from our production department. You will receive proofs shortly before the publication date. Only essential corrections can be made at the proof stage so if there are any minor final changes you wish to make to the manuscript, please let the journal office know now.

DISTRIBUTION OF MATERIALS:
Authors are required to distribute freely any materials used in experiments published in Life Science Alliance. Authors are encouraged to deposit materials used in their studies to the appropriate repositories for distribution to researchers.

You can contact the journal office with any questions, contact@life-science-alliance.org

Again, congratulations on a very nice paper. I hope you found the review process to be constructive and are pleased with how the manuscript was handled editorially. We look forward to future exciting submissions from your lab.

Sincerely,

Tim Fessenden
Scientific Editor
Life Science Alliance
